# Retention of a SulP-family bicarbonate transporter in a periplasmic N_2_-fixing cyanobacterial endosymbiont of an open ocean diatom

**DOI:** 10.1093/ismejo/wraf202

**Published:** 2025-09-04

**Authors:** Mercedes Nieves-Morión, Rubén Romero-García, Sepehr Bardi, Luis López-Maury, Martin Hagemann, Enrique Flores, Rachel A Foster

**Affiliations:** Department of Ecology, Environment and Plant Sciences, Stockholm University, Svante Arrhenius väg 20A, Stockholm SE-106 91, Sweden; Instituto de Bioquímica Vegetal y Fotosíntesis, CSIC and Universidad de Sevilla, Américo Vespucio 49, Seville E-41092, Spain; Instituto de Bioquímica Vegetal y Fotosíntesis, CSIC and Universidad de Sevilla, Américo Vespucio 49, Seville E-41092, Spain; Department of Ecology, Environment and Plant Sciences, Stockholm University, Svante Arrhenius väg 20A, Stockholm SE-106 91, Sweden; Instituto de Bioquímica Vegetal y Fotosíntesis, CSIC and Universidad de Sevilla, Américo Vespucio 49, Seville E-41092, Spain; Facultad de Biología, Departamento de Bioquímica Vegetal y Biología Molecular, Universidad de Sevilla, Avenida Reina Mercedes s/n, Seville 41012, Spain; Department of Plant Physiology, Institute of Biosciences, University of Rostock, Rostock D-18059, Germany; Instituto de Bioquímica Vegetal y Fotosíntesis, CSIC and Universidad de Sevilla, Américo Vespucio 49, Seville E-41092, Spain; Department of Ecology, Environment and Plant Sciences, Stockholm University, Svante Arrhenius väg 20A, Stockholm SE-106 91, Sweden

**Keywords:** diatom, cyanobacteria, periplasmic endobiont, SulP-family transporter, inorganic carbon uptake, bicarbonate, BicA, Richelia euintracellularis, Richelia intracellularis

## Abstract

Symbioses between diatoms and the N_2_-fixing, heterocyst-forming cyanobacteria *Richelia* spp. are widespread and contribute to primary production. Unique to these symbioses is a variation in the symbiont location: one lives in the host cytoplasm (endobiont) vs. residing between the host frustule and plasmalemma (periplasmic endobiont). Both partners are photosynthetic, yet how the partners acquire, share, or compete for bicarbonate necessary for their photosynthesis is unknown. The genomes of both endobionts (ReuHH01 and RintRC01, respectively) contain genes encoding SulP-family proteins, which are oxyanion transporters. To study the possible involvement of these transporters in bicarbonate uptake, we used complementation in a *Synechocystis* sp. PCC 6803 mutant that is unable to grow at air levels of CO_2_ because all five of its inorganic carbon uptake systems have been inactivated. Of the five genes tested, only one (RintRC_3892) from the periplasmic endobiont complemented the mutant to grow with air levels of CO_2_ or at low bicarbonate concentrations. The complemented strain showed strong sodium-dependent and low-affinity bicarbonate uptake that was consistent with bicarbonate concentrations expected in the diatom periplasm. Additionally, all the amino acids involved in the bicarbonate binding site of BicA from *Synechocystis* sp. PCC 6803 are conserved in RintRC_3892. Finally, the importance of the RintRC_3892 protein was confirmed by the consistent detection of its transcripts in wild *Richelia* populations from three different oceans. Combined our results showed no evidence for a bicarbonate transporter in the cytoplasmic endobiont, whereas the periplasmic endobiont has retained a SulP-type bicarbonate transporter for its own photosynthesis.

## Introduction

Diatoms are a widespread group of unicellular eukaryotic phytoplankton that are responsible for ~20% of the primary production on Earth [1]. Although nutrient availability (e.g., nitrate, phosphate) is often the limiting factor on phytoplankton growth, including diatoms, CO_2_ can be equally scarce. To overcome CO_2_ limitation, diatoms have evolved a unique CO_2_ concentration mechanism (CCM) to increase CO_2_ around ribulose-1,5-bisphosphate carboxylase/oxygenase (RuBisCO) [2], the CO_2_-fixing enzyme in photosynthesis. Accordingly, diatoms actively transport and concentrate HCO_3_^−^ from the surrounding into the cell, where HCO_3_^−^ enters the chloroplast compartment and carbonic anhydrases convert HCO_3_^−^ into CO_2_ that is then fixed by RubisCO housed within the pyrenoid [2]. The elevated CO_2_ concentrations around RubisCO results in a depletion of inorganic carbon (C) in the cytoplasm that leads to a passive diffusion of CO_2_ into the diatom cytoplasm from the environment [2]. Within the cell, carbonic anhydrases can act and convert CO_2_ to HCO_3_^−^, thus further increasing HCO_3_^−^ concentration in the cytoplasm and chloroplast. In accordance with this “chloroplast-pump” CCM model, there is a concentration gradient of HCO_3_^−^ within the cytoplasm and periplasmic space (region between the cytoplasmic membrane and outer silicified cell wall) of the diatom, which has implications for our study here on symbiotic diatoms.

Most diatoms reside in coastal zones where nutrients are high, whereas only a few genera of diatoms, primarily those that live in symbiosis with N_2_-fixing cyanobacteria, are capable of forming large scale blooms in oligotrophic areas of the open ocean [3]. These symbioses have an important role in the biosphere given their high rates of both CO_2_ and N_2_ fixation, high measured export, and C sequestration to the deep ocean [4]. Two important diatom-N_2_-fixing symbioses involve the diatoms *Hemiaulus hauckii* and *Rhizosolenia clevei* that form stable and specific relationships with the heterocyst-forming cyanobacteria *Richelia euintracellularis* and *R. intracellularis*, respectively [5]. The cellular location of the *Richelia* symbionts varies such that in *H. hauckii*, *R. euintracellularis* is a fully integrated endobiont, residing in the diatom cytoplasm [6, 7]. In *Rh. clevei* diatoms, the *R. intracellularis* symbionts are located in the periplasmic space of the diatom [8]. A third diatom-*Richelia* symbiosis exists and involves *R. rhizosoleniae* which is a facultative symbiont that attaches extracellularly (epibiont) to the diatom *Chaetoceros compressus* [5, 9]. The fixation and transfer of reduced N from *Richelia* to their respective hosts has been shown in each of the symbioses [10, 11]. In fact, the *Richelia,* when in symbiosis, fixes in great excess of the N required for their own growth [10], yet how such high rates of N_2_ fixation are sustained is not understood.

Genome sequences are available for all the *Richelia* spp. symbionts (ReuHH01, RintRC01, RrhiSC01) [12, 13]. Similar to other cyanobacteria, all the symbionts possess genes encoding several CCM components such as β-carboxysomes, RubisCO, and carboxysomal carbonic anhydrases, as well as a CO_2_ trapping system homologous to the cyanobacterial NDH-1_3_ complex [14]. Hence, they are predicted to use CO_2,_ which is converted into HCO_3_^−^ by the NDH-1_3_ complex, and subsequently reduced by RubisCO inside the carboxysome. High rates of C fixation have also been reported during high density blooms of both *Hemiaulus*- and *Rhizosolenia-Richelia* symbioses in the wild [3, 11, 15]. Unexpectedly as photosynthetic organisms, genomes of both endobionts (ReuHH01, RintRC01) lack genes for high affinity bicarbonate transporters [14], such as the Na^+^-dependent SbtA protein and the ABC-type Cmp transporter which are common in other cyanobacterial genomes [16, 17], including the facultative *Richelia* symbiont (RrhiSC01). However, both *Richelia* endobionts possess homologues to the SulP-family transporters, which include the low-affinity, Na^+^-dependent bicarbonate transporter BicA, first described in *Synechococcus* sp. PCC 7002 [18]. However, in the true endobiont (ReuHH01), one homologue is fragmented into four consecutive sequences [14]. SulP-family transporters are generally involved in the uptake of anions (frequently oxyanions) such as sulfate, nitrate, molybdate and, relevant here for autotrophy, bicarbonate [19]. Sequence homology is not sufficient, however, to resolve neither their function nor specific substrate.

Here, we addressed whether the SulP-family transporters from the two *Richelia* endobionts, *R. euintracellularis* (ReuHH01) and *R. intracellularis* (RintRC01)*,* could function in inorganic C uptake within their respective symbiosis. Because the diatom-*Richelia* symbioses have evaded long-term cultivation, and the endosymbionts appear unable of independent growth [5], we have used heterologous gene expression to investigate the transporter function and substrate affinity. The recipient organism for expressing the *Richelia* genes is the Δ5 mutant of the cyanobacterial model strain *Synechocystis* sp. PCC 6803, in which all five inorganic C uptake systems are inactivated [20]. We show multiple lines of evidence for the retention of an inorganic C uptake strategy in a periplasmic diatom endobiont (RintRC01) that potentially enables it to compete with its host for bicarbonate.

## Materials and methods

### Strains and general growth conditions

*Synechocystis* sp. strain PCC 6803 wild-type strain [WT] was grown with shaking (100 rpm) at 30°C in a modified liquid BG11 medium which replaces ferric ammonium citrate [21] with ferric citrate in continuous light (ca. 25 to 30 μmol photons m^−2^ s^−1^). BG11 contains 0.189 mM Na_2_CO_3._ The *Synechocystis* Δ5 mutant (Δ*ndhD3/ndhD4/cmpA/sbtA/bicA*) (hereafter Δ5 mutant) [20] is a high CO_2_ requiring mutant and therefore was grown in BG11C_10_ or BG11C_50_ medium (BG11 medium supplemented with 10 mM or 50 mM NaHCO_3,_ respectively) at 30°C, bubbled with 1% (v/v) CO_2_ in air under continuous light (50 μmol photons m^−2^ s^−1^), or in BG11C_10_ medium at 30°C with shaking (100 rpm) under continuous light (ca. 25 to 30 μmol photons m^−2^ s^−1^). The construction of the Δ5 mutant relies on cassettes to inactivate *ndhD3, ndhD4, cmpA, sbtA*, and *bicA* which give resistance to spectinomycin, kanamycin, hygromycin, chloramphenicol, and gentamycin, respectively. Thus, the following five antibiotics were used when growing the Δ5 mutant at the indicated concentrations: streptomycin dihydrochloride pentahydrate (Sp), 5 μg ml^−1^; kanamycin (Km), 10 μg ml^−1^; hygromycin (Hyg), 2 μg ml^−1^; chloramphenicol (Cm), 5 μg ml^−1^; and gentamycin (Gent), 1 μg ml^−1^. Cultures were diluted every 5–6 days. To select for a possible complementing gene, erythromycin (Em) at 10 μg ml^−1^ was added to the BG11C_10_ medium supplemented with the five antibiotics described above. For tests on solid medium, BG11 medium was solidified with 1% (wt/v) purified Difco Bacto agar. The transformants were kept in shaking liquid culture (100 rpm) or on solid culture in the corresponding medium described above. To run the ^14^C-bicarbonate uptake assays and the qPCR analysis, WT, Δ5 mutant and transformants were initially grown in BG11C_50_ medium at 30°C, bubbled with 1% (v/v) CO_2_ in air under continuous light and supplemented with the corresponding antibiotics. The strains were then incubated for 18 h in BG11 medium bubbled with air under continuous light. Chlorophyll *a* (Chl) content of the cultures was determined by methanol extraction [22]. *Escherichia coli* strain DH5α, used for plasmid constructions, was grown in Luria broth (LB) medium, supplemented when appropriate with antibiotics at standard concentrations [23].

### Analysis of DNA and protein sequences *in silico*

The search for *Richelia* spp. sequences and data analysis to prepare constructs was carried out using the Integrated Microbial Genomes (IMG) page of the Joint Genome Institute (https://img.jgi.doe.gov) (Table 1). Clustal W was used for aligning protein sequences (https://www.ebi.ac.uk/Tools/msa/clustalo/) with the following parameters: Output format: clustalW with character counts; Dealign input: no; Mbed-like clustering guide-tree: yes; Mbed-like clustering iteration: yes; Combined iterations: default(0); Max guide tree: default; Max hmm iterations: default; Order: aligned; Distance matrix: no; Output guide tree: yes. The phylogenetic analysis of SulP-like proteins was performed in Phylogeny.fr (http://www.phylogeny.fr) [24] using a maximum-likelihood approach and default values (Supplementary Methods).

**Table 1:** Summary of diatom-*Richelia* symbioses, including the associated host, symbiont cellular location, the candidate SulP-like proteins, results from transformation in the *Synechocystis* Δ5 mutant, and evaluation of codon bias for candidates of this study. The + indicates the results of the expression and/or regain of the phenotype (growth) of the *Synechocystis* Δ5 mutant cell line when transformed with the candidate SulP-like proteins; MILC, measure independent of length and composition, calculates the codon bias of individual genes and predicts the gene expression level; for reference, the average MILC for the *Synechocystis* sp. PCC 6803 (WT) genome is 0.66 and for the respective *bicA* is 0.611. nt, not tested, n/a, not applicable. *, bicarbonate uptake in the corresponding transformant.

**Diatom Host**	***Richelia* spp.**	**Symbiont cell location**	**SulP candidate (bp)**	**Expression in ∆5 mutant/ regain of phenotype**	**MILC**
*Hemiaulus hauckii*	*R. euintracellularis*	endobiont	RintHH_3990–60 RintHH_20770	+/− +/−	0.74 0.73
*Rhizosolenia clevei*	*R. intracellularis*	periplasmic symbiont	RintRC_3409 RintRC_4851	+/− +/−	0.66 0.80
			RintRC_3892*	+/+	0.73
*Chaetoceros compressus*	*R. rhizosoleniae*	epibiont	Ga0265390_10577 Ga0265390_11353 Ga0265390_13451	nt nt nt	n/a n/a n/a

**Figure 1:**
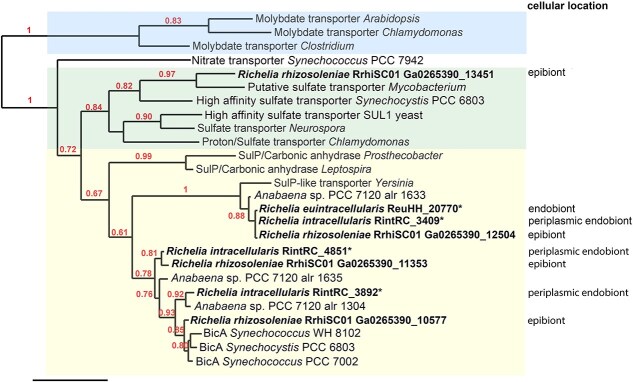
Phylogenetic analysis of SulP-like proteins from *Richelia* spp. that vary in their cellular location with their respective hosts: epibiont, periplasmic endobiont, and cytoplasmic endobiont (noted as endobiont). The homologues from *Richelia* symbionts are highlighted in bold; *Richelia* proteins tested by heterologous expression in this study are designated by an asterisk (*). Phylogenetic analysis was performed in http://www.phylogeny.fr with default values. Shading on the tree is used to distinguish clades: molybdate transporters (top), sulfate transporters (middle), and BicA and SulP proteins associated with carbonic anhydrases likely involved in bicarbonate uptake (bottom). Number of bootstraps, 100. Scale bar is 0.6 substitutions per site. See supplementary methods for gene ID/protein identification.

The bicarbonate binding site has been defined and verified previously for BicA from *Synechocystis* sp. PCC 6803 [25]. Accordingly, we aligned the relevant fragments (~500 residues) of the SulP-like proteins from *Richelia* and *Synechocystis* sp. BicA in order to identify if there was a conservation in the amino acid residues involved in the binding site. We additionally included *Synechococcus* sp. PCC 7002 as its sequence also clustered with the *Richelia* sequences and *Synechocystis* sp. PCC 6803 in the phylogenetic analysis of SulP-like proteins (Fig. 1).

The codon usage and prediction of gene expression for the five target symbiotic genes and the genome of *Synechocystis* sp. PCC 6803 were analyzed using MILC, measure independent length and composition, in the R package called coRdon, which is a part of Bioconductor [26].

### Construction of plasmids containing the symbiont genes

The five symbiotic genes were cloned in plasmid pNRSD_P*_cpcB_*_Ery [27] containing sequences of the *nrsD* gene as DNA fragments for recombination, the promoter of *cpcB* (encoding C-phycocyanin beta subunit) and an erythromycin resistance cassette. The five selected symbiotic genes were synthesized with 100% sequence verification by Integrated DNA Technologies (IDT, Leuven, Belgium) and provided in a pUCIDT plasmid. RintHH_3990–60, RintHH_20770, and RintRC_3409 were digested with BamHI from the pUCIDT plasmids and cloned into pNRSD_P*_cpcB_*_Ery, producing pMN21, pMN22 and pMN24, respectively. RintRC_4851 was digested with NdeI and XhoI from pUCIDT and cloned into pNRSD_P*_cpcB_*_Ery, producing the pMN23. RintRC_3892 was PCR-amplified from a synthetic RintRC_3892 sequence using primers RintRC_3892–5 and RintRC_3892–6 with NdeI and SphI restriction enzymes and cloned into pNRSD_P*_cpcB_*_Ery producing the plasmid pMN10. To verify the constructs and select the correct gene orientation, which is the same orientation as that of the *cpcB* promoter, PCR was carried out using forward primer P*_cpcB560_*_F_EcoRI and specific reverse primers for each gene: RintHH_3990–1, RintHH_20770–1, RintRC_3409–1 and RintRC_4851–1, RintRC_3892–2 (Table S1). The final constructs were verified by sequencing analysis.

### Transformation of *Synechocystis* Δ5 mutant

To attempt complementation with the symbiotic genes, the *Synechocystis* Δ5 mutant was transformed with the plasmids carrying the genes of interest (pMN21, pMN22, pMN24, pMN23, and pMN10, respectively). To do the transformation, optical density at 750 nm (OD_750nm_) of the *Synechocystis* Δ5 mutant cell suspension was set up between 0.6 and 1.0. Mutant cells were washed and resuspended with BG11C_10_ medium, and 200 μl of cell suspension was mixed with 20 μl of plasmid preparation (~200 ng DNA) obtained using a commercial kit (QIA prep Spin Miniprep Kit, Qiagen) and incubated in darkness overnight at room temperature. After that, transformed cells were incubated with 1 ml of BG11C_10_ medium for 4 h at 30°C under light (5–10 μmol photons m^−2^ s^−1^). Subsequently, the cells were placed into BG11C_10_ agar plates for 48 h at 30°C under similar light conditions. Transformed cells were then selected with Em at 10 μg Chl ml^−1^. Em-resistant clones were isolated and their genetic structure was verified by PCR. *Synechocystis* strains bearing the corresponding construct were named RintHH_3990–60, RintHH_20770, RintRC_3409, RintRC_4851, and RintRC_3892, respectively.

### Growth tests on solid medium

For tests of growth on solid medium, *Synechocystis* sp. PCC 6803 (wild-type strain [WT], used as control), the *Synechocystis* Δ5 mutant (used as negative control) and the *Synechocystis* Δ5 mutant transformed with constructs carrying RintHH_3990–60, RintHH_20770, RintRC_3409, RintRC_4851, or RintRC_3892 were grown in liquid cultures of BG11C_10_ medium with the appropriate antibiotics (at final concentration as described above) and air levels of CO_2_ in a shaker (100 rpm) and in continuous light (ca. 25 to 30 μmol photons m^−2^ s^−1^). The cultures of the WT, Δ5 mutant and the transformed *Synechocystis* Δ5 mutants carrying the *Richelia* genes were centrifuged and washed three times with BG11_0_ medium (without any source of nitrogen and without added bicarbonate). The pellets were resuspended in BG11 medium and dilutions containing 1, 0.5, 0.25, 0.125, and 0.0625 μg Chl ml^−1^ were prepared. A 10-μl aliquot of each dilution was spotted on plates with BG11-agar or BG11-agar supplemented with 100 mM of NaHCO_3_ without antibiotics. The plates were incubated at 30°C in continuous light (25–30 μmol photons m^−2^ s^−1^) for 12 or 6 days, respectively.

### Growth tests in liquid medium

The growth rate constant (μ = [ln2]/t_d_, where t_d_ is the doubling time) was calculated from the increase in the optical density at 750 nm (OD_750nm_) of bubbled and shaken liquid cultures. Cultures of *Synechocystis* sp. PCC 6803 (WT, control), the *Synechocystis* Δ5 mutant (used as negative control), and the *Synechocystis* Δ5 mutant transformed with constructs carrying RintHH_3990–60, RintHH_20770, RintRC_3409, RintRC_4851 or RintRC_3892 were inoculated at 0.1 μg Chl ml^−1^. Growth rate constants were determined under different conditions based on the NaHCO_3_ concentration: (i) 0, 1 and 10 mM in cultures bubbled with air levels of CO_2_ at 30°C in the light (50 μmol photons m^−2^ s^−1^); (ii) 1 and 10 mM in shaken cultures (100 rpm) with air levels of CO_2_ at 30°C in the light (ca. 25–30 μmol photons m^−2^ s^−1^). Cultures were supplemented with the appropriate antibiotics at the concentration described earlier.

### ^14^C-bicarbonate uptake assays

To test bicarbonate transport in the *Synechocystis* strains, a ^14^C-bicarbonate uptake assay was performed. Bicarbonate uptake was routinely determined in 1-min assays with the indicated concentration of [^14^C]NaHCO_3_ in BG11 medium or in a buffer, with air-levels of CO_2_ and supplemented with NaCl (concentrations specified below), at 30°C and in the light (75 μmol m^−2^ s^−1^). Cells (10 μg Chl ml^−1^) that had been grown as indicated earlier were incubated for 18 h in BG11 medium bubbled with air under continuous light (50 μmol photons m^−2^ s^−1^). To test the bicarbonate uptake at varying pH values: 6.0, 7.0, and 9.3, cells of *Synechocystis* WT and the strains carrying the *Richelia* genes, were prepared as described above and incubated with 1 mM [^14^C]NaHCO_3_ in BG11/2 medium (2-fold dilution of BG11 medium) with 25 mM TES-KOH (pH 7), MES-KOH (pH 6), or Bis-Tris-propane-HCl (pH 9.3) buffer with a final Na^+^ concentration of ~33 mM. Solutions of ^14^C-labeled bicarbonate for the uptake assays were prepared by mixing unlabeled NaHCO_3_ and [^14^C]NaHCO_3_ at 55 mCi/mmol (American Radiolabeled Chemicals, Inc., MO USA). The additions of [^14^C]NaHCO_3_ were 8–9% of the ambient concentration of HCO_3_. In every ^14^C tracer experiment, the assay was started by mixing cells and substrate. After 1 min (or different times in the experiment when indicated), the assay was finalized by a 25- to 50-fold dilution in ice-cold medium or buffer, the cells were harvested by filtration onto 25 mm diameter 0.45-μm pore size filters (Membrane Filter Millipore) and the filters were washed three times with 2 to 3 ml of ice-cold medium or buffer, and placed in Ecolite(+) scintillation cocktail. The radioactivity in the filters carrying the cells was then determined on a Beckman Coulter LS6500 Liquid Scintillation Counter; boiled cells were used as a blank.

Given the uptake results of the Δ5 mutant complemented with RintRC_3892, we further examined uptake kinetics of this transformant and the necessary controls (*Synechocystis* WT, Δ5 mutant) in a series of conditions. To study Na^+^ dependency (provided as NaCl) on the uptake of 0.11 mM [^14^C]NaHCO_3_ in *Synechocystis* WT and the Δ5 mutant complemented with RintRC_3892, cells prepared as described above were resuspended in 20 mM TES-KOH buffer, pH 8 and tested at the following Na^+^ concentrations: 0.1, 2.1, 4.1, 10.1, 20.1, 30.1, and 40.1 mM. To investigate the effect of the concentration of NaHCO_3_ on the uptake [^14^C]NaHCO_3_ in *Synechocystis* WT, Δ5 mutant and Δ5 mutant complemented with RintRC_3892, cells prepared as described above were resuspended in 20 mM TES-KOH buffer, pH 8, and supplemented with 30 mM NaCl.

### qPCR analyses of expression of *Synechocystis bicA* and *Richelia* genes in the complemented Δ5 mutant

To verify expression of the complemented genes, qPCR analysis was performed on RNA isolated from WT *Synechocystis*, Δ5 mutant complemented with the different *Richelia* genes (RintRC_3892, RintRC_4851, RintRC_3409, RintHH_20770, RintHH_3990–60) and the Δ5 mutant as a control. The WT, Δ5 mutant, and transformant strains were grown as described earlier until a density of ~10 μg Chl ml^−1^. Cells were harvested by gentle filtration (0.45-μm pore size filters, Membrane Filter Millipore) and washed with TE_50_/EDTA_100_. The filter was placed in a tube containing 400 µl of RLT lysis buffer provided in the RNeasy Plant Mini Kit (Qiagen, Germany), amended with 1%β-mercaptoethanol and glass beads. To disrupt the cells, samples were vortexed for 2 min and centrifuged (15 000 × *g*, 5 min, 4°C). Supernatant was collected and an equal volume of cold ethanol was added. Samples were treated as according to kit protocol. RNA was treated with Ambion Turbo DNA-free DNase (Invitrogen) according to the manufacturer’s protocol. The RNA was quantified using a NanoDrop (Thermo Scientific). Three independent RNA samples were analyzed from each strain. RNA (300 ng) was reverse transcribed (RT) to cDNA using a QuantiTect Reverse Transcription Kit with random hexamer primers following the standard protocols of the manufacturer. The reactions were stored at −20°C. TaqMAN quantitative real-time PCR assays (see below) were performed using 2 µl of the cDNA as template and specific oligonucleotides to detect *bicA* (*sll0834*) and *secA* (*sll0616*) genes of WT *Synechocystis* and the various *Richelia* genes in the cDNA (Table S2). The *secA* gene was used for normalizing gene expression in the WT, Δ5 mutant, and Δ5 mutant complemented with various *Richelia* genes.

### Environmental sampling and gene expression

Thirty-one whole water field samples (2–3 L) were collected and filtered with a peristaltic pump onto a 0.2 µm pore size Supor filter (Pall Corporation) held within 25 mm diameter Swinnex filter holders (Millipore) for bulk RNA extraction from several locations in the North Atlantic (NA) and South China Sea (SCS) (Table S3) (further details in Supplementary Methods). The large difference in filtration volumes (0.5-3 L) is related to the conditions at the time of sampling, e.g., particulate loading in the SCS due to some stations at a closer proximity to the Mekong River outflow. Total RNA was extracted using Qiagen RNAeasy Mini kit (Qiagen) following the manufacturer’s protocol with a few modifications described previously [9]. All samples were subjected to a 1-hour DNAse treatment (Qiagen RNase-free DNase kit), and final elution volume was 20 µl. RNA was quantified using Quant-it RiboGreen (ThermoFisher) quantification kit following the kit instructions. Two to 5 µl of total RNA was RT using a commercially available kit (Superscript First Strand Synthesis System). Initially we normalized the template addition for the RT reactions by diluting the total RNA to 5 ng μl^−1^; however, this resulted in a majority of the qPCRs as below detection (bd). Furthermore, as the total RNA template and subsequent cDNA was limited in volume and in order to run all three assays with triplicate pseudo-replicates and accounting for the No-RT controls, we had to modify the volume of cDNA (0.5–2 µl) used as template in the PCRs. Three newly designed TaqMan quantitative polymerase chain reaction (qPCR) assays were designed to quantify the expression of the two *bicA* candidates: RintRC_3892 and RintRC_4851, and in order to normalize the expression, a *secA* (RintRC_6009) assay was designed. The target-specific TaqMAN (Applied Biosystems) oligonucleotides were designed and synthesized by IDT (Table S2; Supplementary Methods). Each probe was 5’ labelled with the fluorescent reporter FAM (6-carboxyfluoreceom) and 3′ labelled with TAMRA (6-carboxytetramethylrhodamine) as a quenching dye. All qPCRs contained 12.5 µl of 2x TaqMAN buffer (Applied Biosystems), 10 µl of molecular biology grade water, 1.0 µl each of forward and reverse primers (10 µM), 0.5 ml probe (10 µM), and 1–2.0 µl cDNA template or separately 2 µl of water for no template controls and 2 µl of standards. Expression was quantified using duplicate standard curves of synthesized gBlocks (IDT) for each target made in an 8-point dilution series (10^8^ to 10^1^ gene copies per reaction) and run in parallel with all samples. Samples were run in triplicate reactions for cDNA and gene copies calculated based on the average cycle threshold value and the standard curve for the appropriate assay. Samples that had one or two of the three replicates detected are reported as detected, not quantifiable (dnq); the limit of detection for the qPCR assays is 1–10 copies. Further details on the design and testing the specificity of all oligonucleotides used in this study are described in full detail in the Supplementary Methods (Table S2; Supplementary Fig. S11).

Gene expression profiles of the three SulP-like proteins from *R. intracellularis* RC01 (RintRC_3892, RintRC_4851, and RintRC_3904), were identified in a publicly available metatranscriptome dataset from the North Pacific Subtropical Gyre [28]. The expression profiles for the above-mentioned SulP-like proteins are provided in the supplementary datasets (Supplementary Table S2 in [28]) and the genes were verified by using their original feature IDs as search queries in the Bacterial and Viral Bioinformatics Resource Center [29]. After verification the data was plotted in R [30]. The meta-transcriptome dataset included results from non-parametric rhythmicity analyses (RAIN), e.g., indicating whether there was significant periodic (diel) gene expression.

## Results

### Several SulP-like proteins are encoded in *Richelia* spp. genomes

Using BlastP analyses, we identified that the number of genes encoding SulP-like proteins varied in number and completeness in the three *Richelia* spp. genomes. Four, three and one complete gene were found in the facultative *Richelia* symbiont (RrhiSC01), periplasmic *Richelia* endobiont (RintRC01), and cytoplasmic *Richelia* endobiont (ReuHH01), respectively (Supplementary Fig. S1 & Fig. S2). To gain insight into the possible function of these *Richelia* SulP-like proteins, a phylogenetic analysis was carried out that included a representative number of non-metazoan SulP proteins [19].

The SulP-like proteins encoded in the *Richelia* spp. genomes clustered in a group of BicA and SulP proteins associated to carbonic anhydrases that are likely involved in bicarbonate uptake [31]. The latter group segregated with good bootstrap support from known sulfate and molybdate transporters (Fig. 1), as well as from one unusual cyanobacterial nitrate transporter [32]. Only one candidate SulP-like protein from the facultative symbiont (RrhiSC01) clustered with a bacterial putative sulfate transporter (from *Mycobacterium*; Fig. 1). Because we were interested in identifying possible bicarbonate transporters in the two *Richelia* endobionts, we focused experimentation on the three complete genes/proteins from RintRC01 and the two genes/proteins, one complete and a second that is split into four fragments, from ReuHH01 (Supplementary Fig. S1). All were considered homologues to proteins in the SulP group that contains bicarbonate transporters (Fig. 1).

The crystallographic structure of BicA from *Synechocystis* sp. PCC 6803 is available and its bicarbonate binding site has been defined [25]. This allowed us to check the possible conservation of key amino acid residues of the bicarbonate binding site. Two of the three genes/proteins from the periplasmic *Richelia* endobiont, RintRC_3982 and RintRC_4851, conserve all six amino acids involved in binding of bicarbonate, as is also the case for BicA from *Synechococcus* sp. PCC 7002 (marked in red and blue in Supplementary Fig. S3). The other SulP-like proteins from the *Richelia* endobionts, including the fragmented protein of ReuHH01 (RintRC_3409, RintHH_2070, RintHH_3990-60), conserve only some of those amino acids.

### *Synechocystis* Δ5 mutant is complemented with only one of five candidate SulP homologues: RintRC_3892 from the periplasmic endobiont

**Figure 2:**
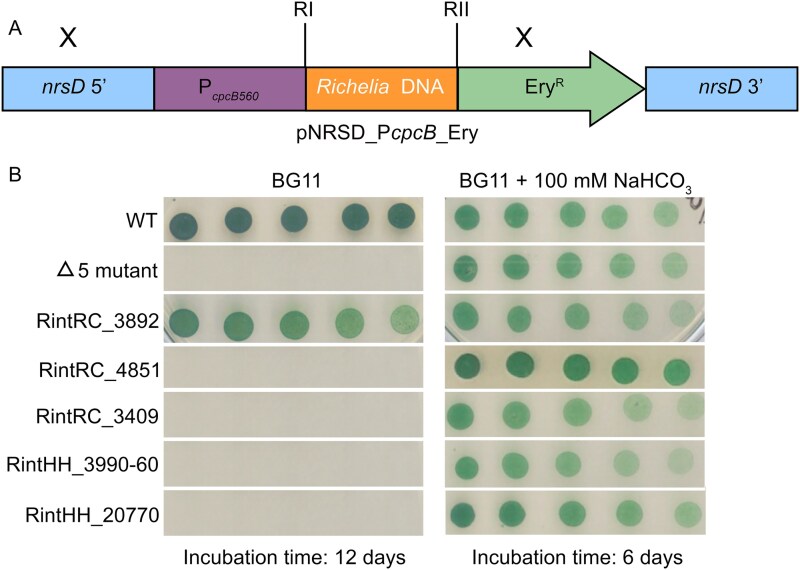
Complementation of *Synechocystis* strain Δ5 with DNA from *Richelia* sp. RintRC01 and ReuHH01. (A) Scheme of the construct used to introduce *Richelia* spp. DNA into the Δ5 mutant strain. The cloned DNA is located downstream from the P*_cpcB560_* promoter and adjacent to an erythromycin-resistance (Ery^R^) gene, and the whole construct is inserted by double recombination in the non-essential *nrsD* gene. The X symbols designate approximate sites of recombination with the *Synechocystis* chromosome. Restriction sites RI and RII are indicated in Fig. S2. (B) Test of photoautotrophic growth of wild-type *Synechocystis* (WT), the Δ5 mutant and derived strains carrying the indicated *Richelia* genes. Strains grown in BG11 medium (in the presence of antibiotics for the mutant and the transformants), as indicated in Materials and Methods, were resuspended in BG11 medium, dilutions were prepared, and a 10-µl portion of each dilution (from left to right, 1, 0.5, 0.25, 0.125, and 0.0625 mg Chl ml^−1^) was spotted on solid BG11 medium (air levels of CO_2_; BG11 medium contains 0.189 mM Na_2_CO_3_) supplemented or not supplemented with 100 mM NaHCO_3_ and incubated under growth conditions for 12 or 6 days as indicated.

To explore the possible role of the *Richelia* proteins in bicarbonate uptake, we tried complementation with each of the five *Richelia* genes (three from RintRC01, two from ReuHH01) in a mutant of *Synechocystis* sp. PCC 6803 that lacks all five CO_2_ and bicarbonate transporters [20]. According to the analysis for codon usage, all five candidates score high for gene expression (MILC values 0.6–0.74) [26]. This mutant, strain Δ5, is characterized by a high CO_2_-requiring phenotype, because it is unable to grow in ambient air levels of CO_2_ [20]. Using the available genomic information, *Richelia* genes were chemically synthesized and transferred to plasmid pNRSD_P*_cpcB_*_Ery, in which they were placed under the control of the strong promoter P*_cpcB560_* upstream of an erythromycin-resistance gene (Fig. 2A; Figs. S4 and S5). The presence of the P*_cpcB560_*-*sulP*-like gene construct in individual clones derived from the transformations was confirmed by PCR (Fig. S6). In addition, RT-qPCR analyses showed the expression of the *sulP*-like genes in the transformants, albeit at different levels (described in detail below; Fig. S7).

The *Synechocystis* Δ5 mutant was routinely grown in liquid cultures of BG11 medium under high inorganic C conditions (see methods). The growth on plates of the *Synechocystis* Δ5 mutant transformed with the different *Richelia* constructs was first tested on solid BG11 medium using *Synechocystis* WT (hereafter WT) and Δ5 mutant as controls (Fig. 2B). With air levels of CO_2_, only the WT and the Δ5 mutant transformed with RintRC_3892 could grow, whereas the Δ5 mutant and all other transformants with either the *Richelia* RintRC01 genes (RintRC_4851, RintRC_3409) or the *Richelia* ReuHH01 genes (RintHH_20770, RintHH_3990-60) failed to grow (Fig. 2B). In contrast, all the strains could grow when the medium was supplemented with 100 mM NaHCO_3_ (Fig. 2B).

Growth of the different strains was also tested in air-bubbled BG11 medium not supplemented or supplemented with 1 or 10 mM NaHCO_3_, and the growth rate constant was determined for the different strains (Table 2; Fig. S8). Without added NaHCO_3_, growth was observed for the WT and only the Δ5 mutant carrying RintRC_3892 (0.55 ± 0.013 day^−1^ and 0.88 ± 0.006 day^−1^, respectively) (Table 2). With 1 mM NaHCO_3_, growth was observed for the WT and again only the Δ5 mutant carrying RintRC_3892 (1.14 ± 0.2 day^−1^ and 1.07 ± 0.06 day^−1^, respectively). In both conditions (0 or 1 mM NaHCO_3_), growth rate constants were significantly higher in the Δ5 mutant carrying RintRC_3892 than for the Δ5 mutant (*P* value <0.001 by Student’s t test; Table 2). In contrast, growth was observed for all strains, including the Δ5 mutant, when supplemented with 10 mM NaHCO_3_. Growth was also tested in shaken cultures of BG11 medium (ambient air levels of CO_2_) at two different NaHCO_3_ concentrations; only the WT and the Δ5 mutant carrying RintRC_3892 grew in the presence of 1 mM NaHCO_3_, whereas all the strains could grow with 10 mM NaHCO_3_ (Fig. S8). Combined the results show that RintRC_3892 encodes a protein that provides the *Synechocystis* Δ5 mutant with the capacity to grow well in liquid or solid medium with ambient air levels of CO_2_ (which at chemical equilibrium should build a low concentration of HCO_3_^−^) or with a low concentration (1 mM) of HCO_3_^−^; i.e., the RintRC_3892 gene complements the high CO_2_-requiring phenotype of the Δ5 mutant.

**Table 2:** Growth test in bubbled liquid cultures and [^14^C]bicarbonate uptake in WT *Synechocystis* and Δ5 mutant transformed with *Richelia* genes from periplasmic (RintRC) and cytoplasmic (RintHH) endosymbionts. Growth was tested in autotrophic cultures with air-levels of CO_2_ and the indicated bicarbonate concentrations and conditions described in Materials and Methods. The growth rate constant was determined from the number of independent cultures shown in parenthesis. Uptake of 1.1 mM H^14^CO_3_^−^ was tested in 1 min-assays performed at 30°C in the light in BG11 medium supplemented with NaCl (final Na^+^ concentration ca. 33 mM), pH 8; mean and standard deviation (SD) are shown from 3–6 replicate experiments (shown in parenthesis). Differences in growth rate or bicarbonate transport of each strain compared to the ∆5 mutant were assessed by the Student’s t test (*, *P* value <10^−5^; all others *P* value *>*0.1).

**Strain**	**Growth rate constant (day** ^**−1**^**) mean ± SD (n)**			[^14^C] **Bicarbonate uptake** **(nmol [mg Chl]**^**−1**^ **min**^**−1**^**)** **mean ± SD (n)**
Growth conditions: medium + amendment	BG11	BG11+ 1 mM NaHCO_3_	BG11+ 10 mM NaHCO_3_	BG11 + NaCl
*Synechocystis* WT	0.55 ± 0.013 (3)*	1.14 ± 0.20 (3)*	0.81 ± 0.103 (3)	1.45 × 10^3^ ± 325 (3)*
∆5 mutant	0.010 ± 0.001 (3)	0.01 ± 0.001 (3)	0.56 ± 0.31 (3)	6.85 ± 8.10 (6)
∆5 + RintRC_3892	0.88 ± 0.006 (3)*	1.07 ± 0.06 (4)*	1.01 ± 0.07 (3)	1.51 × 10^3^ ± 290 (3)*
∆5 + RintRC_4851	0.02 ± 0.001 (3)	0.04 ± 0.01 (3)	0.75 ± 0.05 (3)	24.7 ± 30.2 (6)
∆5 + RintRC_3409	0.04 ± 0.00 (3)	0.01 ± 0.01 (3)	0.67 ± 0.07 (3)	11.5 ± 10.3 (4)
∆5 + RintHH_3990–60	0.04 ± 0.00 (3)	0.020 ± 0.003 (3)	0.68 ± 0.07 (3)	22.3 ± 18.8 (4)
∆5 + RintHH_20770	0.04 ± 0.001 (3)	0.00 ± 0.000 (3)	0.71 ± 0.09 (3)	27.5 ± 25.4 (4)

### [^14^C]bicarbonate uptake assays confirm only *Synechocystis* Δ5 transformed with SulP-like protein RintRC_3892 transports bicarbonate

**Figure 3:**
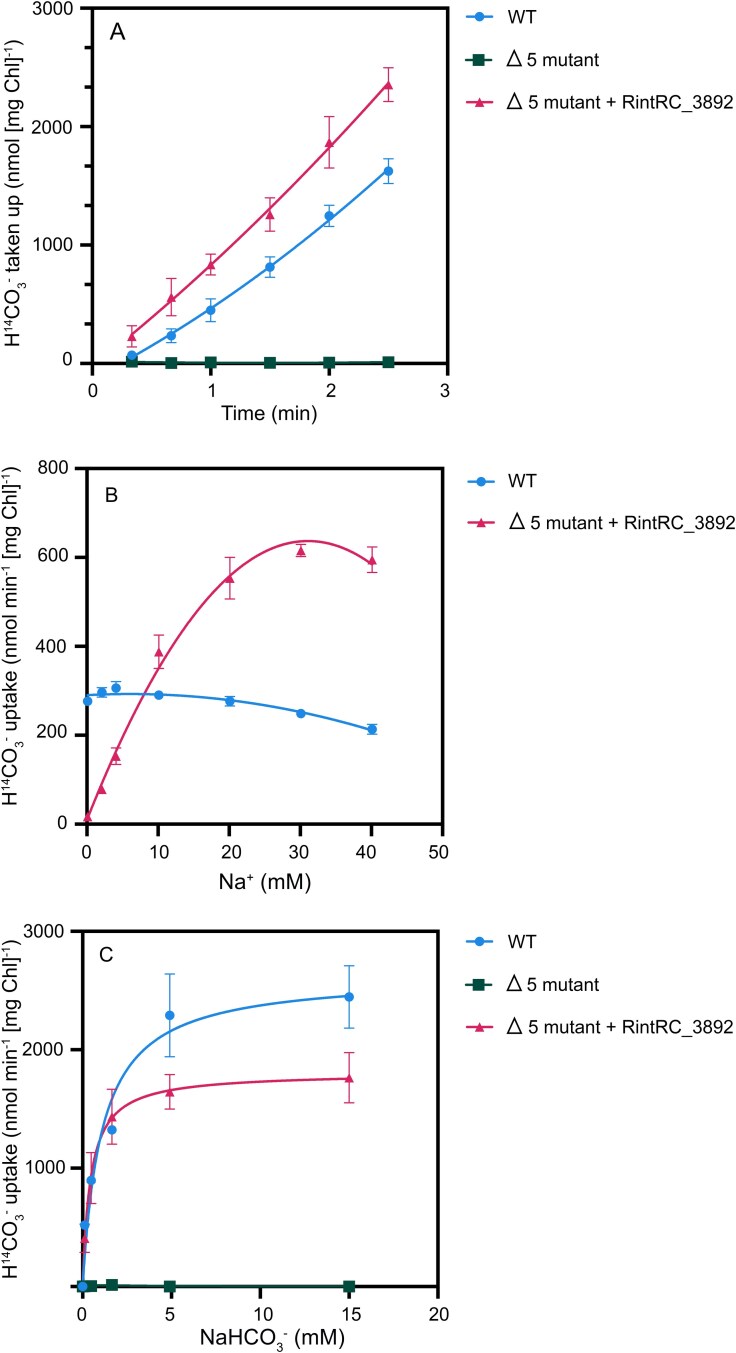
Uptake of [^14^C]bicarbonate by *Synechocystis* WT, the Δ5 mutant, and the Δ5 mutant transformed with RintRC_3892. (A) Uptake was tested with 1 mM bicarbonate (NaH^14^CO_3_) in BG11 medium supplemented with NaCl (final Na^+^ concentration ≈ 33 mM) in cells that had been incubated for 18 h in BG11 medium (with antibiotics for the mutants) with air-levels of CO_2_. Shown are the mean and standard deviation from a representative experiment with three independent cultures and a second order polynomial (quadratic) fitting model, (*R*^2^, WT = 1.00, Δ5 = 0.51, RintRC_3892 = 1.00). (B) Na^+^-dependency of [^14^C]bicarbonate uptake in WT *Synechocystis* and mutant Δ5 complemented with RintRC_3892. Effect of Na^+^ (provided as NaCl) on the uptake of 0.11 mM NaH^14^CO_3_ in cells suspended in 20 mM TES-KOH buffer, pH 8. Shown are the mean and standard deviation from a representative experiment with three independent cultures and a second order polynomial (quadratic) fitting model (*R*^2^, WT = 0.93, RintRC_3892 = 0.99). (C) Bicarbonate-dependency of [^14^C]bicarbonate uptake in WT *Synechocystis* and mutant Δ5 complemented with RintRC_3892. Effect of the concentration of NaHCO_3_ on the uptake NaH^14^CO_3_ in cells suspended in 20 mM TES-KOH buffer, pH 8, supplemented with 33 mM NaCl (final sodium concentration increases above this value with added NaHCO_3_). Shown are the mean and standard deviation from a representative experiment with three independent cultures and a Michaelis–Menten fitting model, (*R*^2^, WT = 0.97, Δ5 = 0.66, RintRC_3892 = 1.00). The assays were carried out and the cells were prepared as described in Materials and Methods. The legends to the right of each graph (A-C) explain the symbols and respective strain.

To test directly transport of bicarbonate, we set up a bicarbonate uptake assay in which cells that had been incubated for 18 h in air-levels of CO_2_ were incubated in BG11 medium or a buffer and supplemented with [^14^C]bicarbonate at 30°C in the light (see Materials and Methods). As observed in the WT, ^14^C-labeled bicarbonate from BG11 medium (pH 8) amended with 1 mM [^14^C]bicarbonate was efficiently taken up, and the rate of uptake progressively increased during a 2-min assay (Fig. 3A). This increase may reflect the time required for homogenization of the solution after mixing cells and substrate, DIC species equilibration and subsequent transport of bicarbonate and trapping of CO_2_. The Δ5 mutant did not show any significant uptake, whereas the Δ5 mutant complemented with RintRC_3892 showed significant activity (*P* value <0.001 by Student’s t test) (Fig. 3A; Table 2). Uptake in the complemented strain was more linear than in the WT, consistent with direct uptake of [^14^C]bicarbonate from the beginning.

To focus on initial transport rates, uptake of ^14^C-labeled bicarbonate was tested in 1-min assays with the different transformed strains. As shown earlier, the WT showed high [^14^C]bicarbonate uptake whereas the Δ5 mutant showed negligible uptake (Table 2). Among the Δ5 strains carrying the *Richelia* genes, the Δ5 mutant carrying RintRC_3892 showed high uptake of [^14^C]bicarbonate similar to WT rates, whereas the other transformants showed only slightly higher but not statistically significant increased uptake than the Δ5 mutant (Table 2).

In order to test whether a more acidic or a more alkaline pH could stimulate the bicarbonate uptake activity, we ran assays at pH 6, 7, and 9.3 resulting in similar results (Fig. S9). An especially high activity at pH 9.3 of RintRC_3892 may result from an increased bicarbonate/CO_2_ ratio at this pH in a strain that has only a bicarbonate transporter. In conclusion, [^14^C]bicarbonate uptake assays are consistent with the results from the liquid growth tests supplemented with 1 mM NaHCO_3_, demonstrating that RintRC_3892 is a fully functional bicarbonate transporter.

### RintRC_3892 is a Na^+^ dependent and low affinity bicarbonate transporter

Because the characterized SulP-family bicarbonate transporter, BicA, is sodium (Na^+^) dependent [18], we determined [^14^C]bicarbonate uptake in the RintRC_3892 complemented Δ5 mutant at a range of Na^+^ concentrations from 0.1 mM to 40 mM. The [^14^C]bicarbonate transport activity showed a strong Na^+^ dependency (Fig. 3B), suggesting that RintRC_3892 mediates a Na^+^:HCO_3_^−^ co-transport as expected for a potential BicA homolog. The Δ5 mutant did not show high [^14^C] bicarbonate uptake when tested at different Na^+^ concentrations: 1.77 ± 0.31 nmol (mg Chl)^−1^ min^−1^ at 33 mM and 6.27 ± 0.41 nmol (mg Chl)^−1^ min^−1^ at 366 mM (four replicates for each assay). On the other hand, the WT showed no Na^+^ dependency (Fig. 3B). It is likely that a significant part of [^14^C]bicarbonate uptake in the WT is mediated by Na^+^-independent transporters such as the ABC transporter Cmp. Given the amino acid conservation of RintRC_4851 with the known bicarbonate binding site (Fig. S3), we tested the Δ5 mutant complemented with RintRC_4851 at both a low and high Na^+^ concentration (33 mM and 366 mM, respectively), but no increased activity was observed (8.51 ± 1.19 and 11.00 ± 1.38, nmol (mg Chl)^−1^ min^−1^, respectively; three replicates for each assay).

We tested the effect of [^14^C]bicarbonate concentration on uptake in the presence of Na^+^ (> 30 mM final concentration). The Δ5 mutant did not show uptake at any concentration tested, whereas the WT showed saturation kinetics likely reflecting the cumulative activity of its several bicarbonate transporters (Fig. 3C). The Δ5 mutant complemented with RintRC_3892 showed saturation kinetics with a *K*_0.5_ value of ~464 ± 108 μM (n = 5 independent cultures). Because RintRC_3892 is the only bicarbonate transporter in this strain, the determined *K*_0.5_ value is an indication of its affinity for bicarbonate. However, this value may be affected by non-labeled bicarbonate present in the solution (e.g., from air CO_2_) with the effect that the actual *K*_0.5_ might be somewhat higher (i.e., lower affinity).

Despite that the WT bears five bicarbonate/CO_2_ transporters, we observed similar or even higher rates of [^14^C]bicarbonate uptake in the RintRC_3892-complemented Δ5 mutant than in the WT (Table 2, Fig. 3). This likely resulted from our strategy to use a very strong promoter (P*_cpcB560_*), which we chose to ensure expression in *Synechocystis* of the cloned *Richelia* genes. qPCR analysis performed on RNA isolated from WT *Synechocystis*, the RintRC_3892-complemented Δ5 mutant and the Δ5 mutant as a control showed that the RintRC_3892-complemented strain expressed RintRC_3892 at levels ~10-fold higher than *Synechocystis* WT expressed *bicA* (normalized cDNA, 68.9 ± 30.3 and 6.73 ± 8.53, respectively) (Fig. S7). This observation helps to understand the high [^14^C]bicarbonate uptake rates attained by the complemented strain with only one transporter. The normalized *bicA*-like transcripts in the other four transformant strains (RintRC_4851, RintRC_3409, RintHH_20770, RintHH_3990–60) were reduced (≈1.2 to 5.7) compared to WT *bicA* (Fig. S7). (The Δ5 mutant is an insertion mutant rather than a deletion mutant and therefore the *bicA* transcripts detected in the Δ5 mutant were produced from a promoter in the antibiotic resistance gene cassette (Fig. S7)).

### *Richelia* RintRC01 SulP-like transcripts are detected in environmental samples

**Figure 4:**
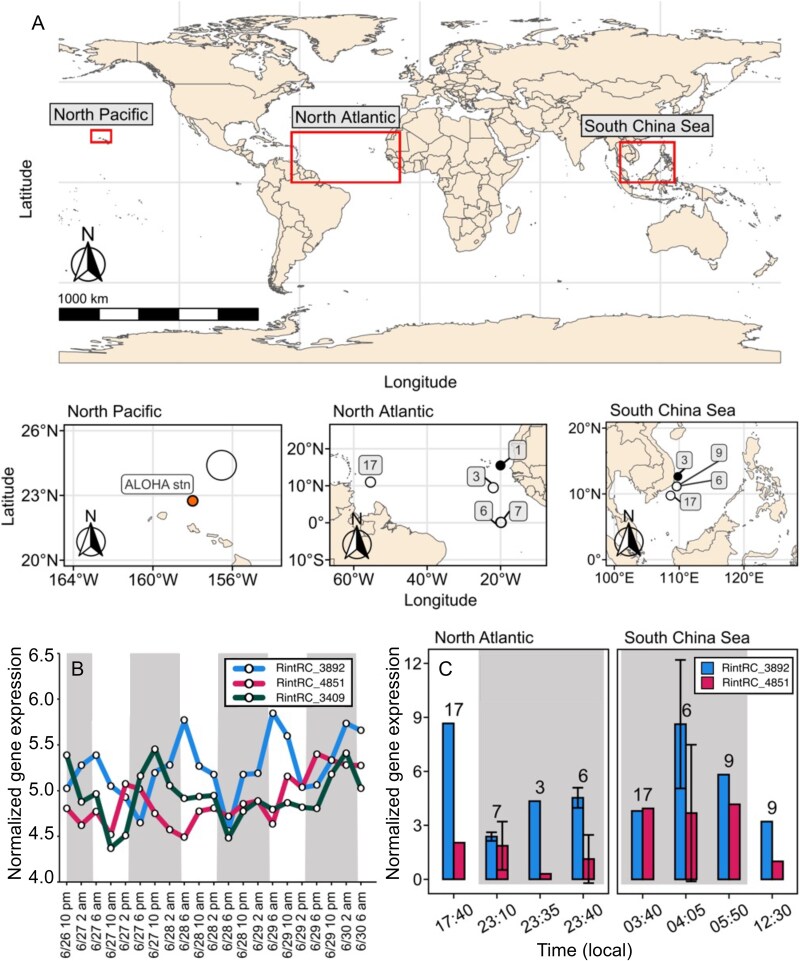
Expression of genes encoding SulP-like proteins of *Richelia* RintRC01 in wild populations. (A) Overview of the global map (top) and locations (below) from which samples for estimating expression of RintRC_3892 and RintRC_4851 by RT-qPCR were collected (North Atlantic, NA; South China Sea, SCS). Transcripts for RintRC_3892, RintRC_4851, and RintRC_3904 were taken from a publicly available transcriptome in the NP. Filled circles indicate stations in NA and SCS where neither SulP-like encoding genes were detected. (B) Diel patterns for normalized gene expression of RintRC_3892, RintRC_4851 and RintRC_3904 from the meta-transcriptome study in the NP [28]. (C) Results of normalized gene expression for RintRC_3892 and RintRC_4851 from stations in the NA and SCS collected at different times; the *secA* gene was used for normalization. The numbers above the bars indicate the station number. For stations which had multiple samples from different depths, the normalized gene expression was averaged (Supplementary Table S3). White and gray boxes in B and C indicate the light and dark periods, respectively.

In order to study the importance of the SulP-homologues in wild populations of *Richelia*, we developed highly specific RT-qPCR assays to quantify gene transcripts of RintRC_3892 and RintRC_4851 in environmental samples (Fig. S10). The assays were applied to bulk RNA samples collected from select locations in two different ocean basins (North Atlantic, NA; South China Sea, SCS; Fig. 4A; Supplementary Table S3) in which field-based microscopy observations of symbiotic *Richelia* RintRC01 were reported previously [33]. Additionally, we mined a publicly available meta-transcriptome from the subtropical North Pacific gyre (Fig. 4B) for identifying the diel expression patterns of RintRC_3892, RintRC_4851, and additionally RintRC_3409 (Supplementary Table S2 in [28]). In the meta-transcriptome dataset, all three SulP-like proteins were expressed; RintRC_3892 showed a significant diel periodicity (RAIN, FDR < 0.05) of highest expression shortly after sunrise (Fig. 4B). Likewise, we consistently detected gene transcripts of RintRC_3892 and RintRC_4851 in both the NA and SCS, and in general, the RintRC_3892 transcripts were usually higher (Fig. 4C).

## Discussion

Oceans are an important reservoir of DIC [34] that is used by marine phytoplankton to perform photosynthesis. In the biological C pump, marine diatoms, especially the diatom-*Richelia* symbioses, make an important contribution to C burial by forming large and expansive blooms which reduce a significant amount of inorganic C that sinks rapidly as particulate C [35–38]. The *Richelia* also fix high amounts of N_2_, which not only fulfills their own and host N growth requirements [10], but additionally serves to release substantial fixed N to the surrounding community, thus supporting primary (new) production. How the *Richelia* symbionts sustain such high rates of N_2_ fixation is an important and open question. In heterocyst-forming cyanobacteria, energy derived from photosynthesis in the vegetative cells supports the N_2_ fixation in the heterocysts [39]. The concentration and transport of inorganic C in the *Richelia* symbionts is therefore essential to consider. Moreover, because of their varying cellular locations (cytoplasm, periplasmic, external), the *Richelia* symbionts also poses an interesting conundrum with whether they compete, collaborate, or possibly help to concentrate inorganic C with their respective hosts.

In the oceans, the concentration of CO_2,_ which is the substrate for the C fixation enzyme, RuBisCO, is very low (<1%) compared to other forms of inorganic C (e.g., HCO_3_^−^, CO_3_^2−^) [34]. Thus, microalgae, including diatoms, have evolved mechanisms (e.g., CCM) for acquiring CO_2_. A phytoplankter cell size and morphology will also impact the availability, competitiveness, and diffusion of nutrients at their cell surface. Large diatoms have larger diffusive boundary layers (DBL) and therefore rely more on active transport of HCO_3_^−^ and, in some, extracellular carbonic anhydrases (eCAs) to overcome the diffusive limitation of their DBL. The presence of eCAs in diatoms is not uniform; e.g., it is considered ubiquitous in centric diatoms, but variable in pennate diatoms [40]. *Rh. clevii*, the host of *R. intracellularis*, are large (20–600 µm) centric diatoms, whereas *H. hauckii*, which hosts *R. euintracellularis*, are comparatively smaller (15–30 µm) pennate diatoms. The biometric relationship that has been reported for the diatom-*Richelia* symbioses, where larger cells have higher C and N_2_ fixation rates [11], suggests that an efficient inorganic C acquisition is necessary. Recent evidence in diatoms suggests that cell size and photosynthetic rate are important components leading to an increased reliance on eCAs [40]. It is therefore interesting to speculate whether, e.g., the *Rhizosolenia* hosts that associate with RintRC01 might also utilize their periplasmic symbionts as an additional CCM.

The *Richelia* spp. genomes possess the necessary genes for photosynthesis, and therefore both partners are photosynthetic. Moreover, high rates of C fixation have been reported when the diatom-*Richelia* symbioses are present [3, 11, 41]. Herein we focused on the identification, functionality, and characterization (e.g., Na^+^ dependency, affinity) of transporters which could mediate the uptake of bicarbonate by the endosymbionts that reside in the cytoplasm and the periplasm of diatoms, i.e., the SulP-family transporters of *R. euintracellularis* HH01 and *R. intracellularis* RC01, respectively. We initially hypothesized that the true endobiont (ReuHH01) likely differed in inorganic C transport strategies given its cytoplasmic location, reduced genome size, presence of fragmented putative transporter genes and recent evidences that it relies more on its host for organic C [12–14, 42]. A major motivation for the work presented here was to circumvent the need for isolated cultures, which for the diatom-*Richelia* symbiosis has not been possible over the long-term [43], and rely on heterologous expression in an appropriate mutant to verify the function of potential bicarbonate transporters.

We found initially that SulP-like proteins vary in number and completeness in the *Richelia* spp. genomes. This result is not unexpected given the varying states of integration of the symbionts with their hosts, and the necessity for inorganic C transport in relation to the host diatoms CCM (Fig. S11). Our results have unequivocally shown that the genome of *R. intracellularis* RC01 [13], which is the periplasmic symbiont of *Rh. clevei*, bears a gene (RintRC_3892) encoding a SulP-type bicarbonate transporter. The affinity of this transporter for bicarbonate (*K*_0.5_ ~464 μM or higher) is relatively low compared to that of high affinity bicarbonate transporters of other cyanobacteria, such as the ABC transporter Cmp (*K*_D_ for bicarbonate of the CmpA protein, 5 μM; [44]) or the Na^+^-dependent monocomponent SbtA transporter (K*_m_* for bicarbonate ~2 μM in *Synechococcus* sp. PCC 7002; 18). However, the affinity of RintRC_3892 for bicarbonate is in the range of the *Synechcoccus* sp. PCC 7002 BicA, which is a known low-affinity but high-flux transporter (K*_m_*, 217 μM) [18]. In relevance to the symbiosis, the affinity of RintRC_3892 is consistent with the concentration of bicarbonate expected in the periplasm of diatoms (~2 mM) [2, 45] (Fig. S11). Given that RintRC01 lacks genes for high-affinity HCO_3_^−^ transporters common to many other cyanobacteria, including the facultative epibiont *Richelia* RrhiSC01, possessing a low-affinity SulP-type transporter could be sufficient to compete with its host for bicarbonate. Alternatively, RintRC01 could be working with its respective host to facilitate HCO_3_^−^ transport given the larger cell size of the *Rh. clevei* hosts, larger DBL, and corresponding higher CO_2_ demand for photosynthesis.

The sodium dependency of RintRC_3892 is also similar to that of *Synechococcus* sp. PCC 7002 BicA (compare Fig. 3B to data in [18]). These observations are consistent with the close phylogenetic position of RintRC_3892 and BicA from different cyanobacteria, providing further evidence that RintRC_3892 from the periplasmic endobiont is a genuine BicA protein. All the amino acids involved in bicarbonate binding in BicA from *Synechocystis* sp. PCC 6803 [25] are conserved in RintRC_3892. And finally, we consistently detected the expression of RintRC_3892 in environmental samples from two ocean basins (NA and SCS), and in the case of the North Pacific (NP) meta-transcriptome study [28], its expression pattern follows a diel periodicity which is expected for an actively photosynthesizing organism.

In neither genomes of *Richelia* endobionts did we identify any homologues of high affinity bicarbonate transporters, Cmp or SbtA [12–14]. Our finding that the genome of *R. intracellularis* RC01 encodes a BicA protein, which shows relatively low affinity for bicarbonate, is therefore of interest, because this transporter is well suited to take up concentrations of bicarbonate (~2 mM) in the marine environment. In contrast, the possible lack of any bicarbonate transport protein in *R. euintracellularis* ReuHH01 might reflect a relatively low availability of bicarbonate in the cytoplasm of the diatom [2] or that ReuHH01 is less active for photosynthesis. These latter results are consistent with the diatom host (i.e., *Hemiaulus*) supporting the physiology of the cytoplasmic symbiont (ReuHH01) with organic C [11, 42].

Our results failed to show complementation of the Δ5 mutant or significant bicarbonate uptake activity by the other tested *Richelia* SulP-like proteins, two from RintRC01 and two from ReuHH01, including a fragmented protein. In the latter four transformants, expression of their respective *bicA*-like genes was also significantly reduced compared to the WT and the one transformant RintRC_3892 which was active for bicarbonate transport. Therefore, our results do not provide evidence that these four proteins function in bicarbonate transport, which is consistent with the lack of conservation of some amino acids in the bicarbonate-binding site for proteins other than RintRC_3892 (which we show is a bicarbonate transporter) and RintRC_4851. The use of a strong promoter could have a measurable impact on gene expression, and likely influenced the high growth rate and bicarbonate transport in the transformant with *Richelia*_3892 compared to the WT, but these results do not impact the overall conclusions of our presented work. We cannot discount, however, that RintRC_4851 functions in bicarbonate transport either under different conditions than those tested in this work or if a higher expression of the gene could be achieved. Alternatively, having conserved the bicarbonate binding site but not mediating bicarbonate transport could suggest a role for RintRC_4851 in binding bicarbonate for other purposes, such as signaling as suggested for permease-homolog proteins in other systems (see, e.g. [46, 47]). Evidence of the importance of RintRC_4851 is found in our field expression analyses as it was consistently detected in all three environmental datasets and showed a higher expression during the late photoperiods. Additionally, RintRC_3409, which only conserved some amino acids for binding bicarbonate, was also identified in the field studies with an obvious dynamic pattern of higher expression in the middle of the dark periods [28], but its actual substrate remains unknown.

Unlike the two endosymbionts studied here, the external facultative symbiont *R. rhizosoleniae* RrhiSC01 contains a high affinity bicarbonate transporter (SbtA), which is not present in the endosymbionts [14]. It can be suggested that this additional transporter is involved in bicarbonate uptake to circumvent the possible competitive situation of living in the phycosphere of its diatom host or when living unattached and free. In summary, our results are consistent with the hypothesis that these cyanobacterial symbionts differ in their C metabolic dependency with their respective hosts as a result of their adaption to a symbiotic life. In the case of the cytoplasmic endobiont (ReuHH01) and recently demonstrated [42], these symbionts have an increased dependence on organic C provided by their hosts whereas the other endobiont (RintRC01) in its periplasmic residence has retained a SulP-type bicarbonate transporter for its own photosynthesis.

## Supplementary Material

Suppl_Figs_and_tables_final_28Aug25_wraf202

## Data Availability

The data that support the findings of this study are available in the article and its Supporting Information.
