# Supplementary material for: Retention of a SulP-family bicarbonate transporter in a periplasmic N2-fixing cyanobacterial endosymbiont of an open ocean diatom
Source: ISME J. 2025 Sep 4;19(1):wraf202. doi: 10.1093/ismejo/wraf202 (PMC12596568; doi:10.1093/ismejo/wraf202)
Supplement: Suppl_Figs_and_tables_final_28Aug25_wraf202 [file suppl_figs_and_tables_final_28aug25_wraf202.pdf]

Retention of a SulP-family bicarbonate transporter in a periplasmic N_2-fixing cyanobacterial endosymbiont of an open ocean diatom

Mercedes Nieves-Morión[1,2]*, Rubén Romero-García[2], Sepehr Bardi[1], Luis López-Maury[2,4], Martin Hagemann[3], Enrique Flores[2], and Rachel A. Foster[1*]

[1]Department of Ecology, Environment and Plant Sciences, Stockholm University, SE-106 91 Stockholm, Sweden; [2]Instituto de Bioquímica Vegetal y Fotosíntesis, CSIC and Universidad de Sevilla, Américo Vespucio 49, E-41092 Seville, Spain; [3]Department of Plant Physiology, Institute of Biosciences, University of Rostock, Rostock, D-18059, Germany. [4]Departamento de Bioquímica Vegetal y Biología Molecular, Facultad de Biología, Universidad de Sevilla, Avenida Reina Mercedes s/n, 41012 Seville, Spain.

*Correspondence: mercedes.nieves@ibvf.csic.es, rachel.foster@su.se

Content of each file:

Figure S1. SulP-like proteins from *Richelia* spp.
Figure S2. Clustal W alignment.
Figure S3. Amino acids in the bicarbonate binding site of BicA.
Figure S4. Cloning strategy to express *Richelia* genes in the *Synechocystis* Δ5 mutant.
Figure S5. DNA sequence covering the RintHH_3960-3970-3980-3990 *sulP*-like gene fragments.
Figure S6. PCR analysis showing the presence of the *Richelia* constructs in the *Synechocystis* Δ5 mutant transformants.
Figure S7. Summary of qPCR analysis of expression of *bicA*.
Figure S8. Growth of WT *Synechocystis* and the transformed Δ5 mutant in air levels of CO_2 supplemented or not with bicarbonate.
Figure S9. Uptake of ^{14}C-bicarbonate at pH 6, pH 7 and pH 9.3 by WT *Synechocystis* and the Δ5 mutant transformed with the indicated constructs.
Figure S10. Summary of the specificity testing of the oligonucleotides used in the RT-qPCR assays for estimating the expression of the SulP-like transporters in field samples by BLASTn analyses (details of databases in Supp. Methods).
Figure S11. Overview of the inorganic C acquisition in the two diatom-*Richelia* symbioses.
Table S1. List of the oligonucleotides used in the construction of plasmids containing the symbiotic *Richelia* genes.
Table S2. Summary of oligonucleotides used in the RT-qPCR assays.
Table S3. Summary of results from the RT-qPCR assays to estimate the expression of RintRC_3892 and RintRC_4851.

Fig. S1. SulP-like proteins from *Richelia* spp. (*top*) BlastP analysis was performed using as query the SulP-type bicarbonate transporter BicA of *Synechococcus* sp. PCC 7002 or, when indicated, the SulP-like protein Alr1633 from *Anabaena* sp. PCC 7120. BlastP expect values are shown in parenthesis. (*Bottom*) Scheme showing the RintHH_3960-3970-3980-3990 genomic region.

**Homologues in *R. rhizosoleniae* SC01**	**Homologues in *R. intracellularis* RC01**	**Homologues in *R. euintracellularis HH01***
Ga0265390_10577<br>(0.0)<br><br>Ga0265390_11353<br>(0.0)<br><br>Ga0265390_12504<br>(1e^{-18})<br><br>Ga0265390_13451<br>(6e^{-44})<br>(0.0, compared to Alr1633)	RintRC_3892<br>(0.0)<br><br>RintRC_4851<br>(0.0)<br><br>RintRC_3409<br>(0.0, compared to Alr1633)	RintHH_3960<br>(4e^{-12})<br>RintHH_3970<br>(e^{-103})<br>RintHH_3980<br>(3e^{-21})<br>RintHH_3990<br>(5e^{-32})<br><br>RintHH_20770<br>(5e-43)<br>(0.0, compared to Alr1633)

Gene possibly split into four fragments

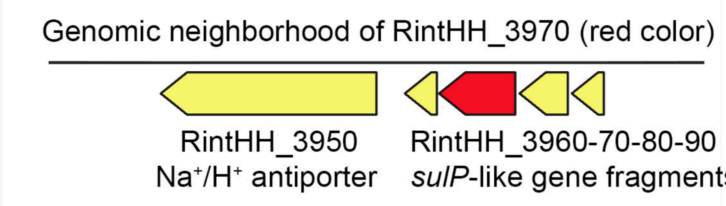

Fig. S2. Clustal W alignment. Alignment of identified *Richelia* SulP-family proteins and their respective queries from *Synechococcus* sp. PCC 7002 or *Anabaena* sp. PCC 7120. (**A**) Alignment of BicA from *Synechococcus* (Ga0265390_10577) and RintRC_3892. The following parameters are used in the alignments: Output format: clustalW with character counts; Dealign input: no; Mbed-like clustering guide-tree: yes; Mbed-like clustering iteration: yes; Combined iterations: default(0); Max guide tree: default; Max hmm iterations: default; Order: aligned; Distance matrix: no; Output guide tree: yes. The following symbols indicate residue conservation: * = identical residues; : = strong similarity; . = weak similarity; (blank) = no conservation. https://www.ebi.ac.uk/Tools/msa/clustalo/

A

```
Synechococcus      MQITNKIHFRNIRGDIFGGLTAAVIALPMALAFGVASGAGAEAGLWGAVLVGFFAALFGG      60
RintRC3892         MQLLNRIHFRNLRGDIFGGLTAAIISLPLALAFGVASGAGPIAGLYGAVIIGLFAALFGG      60
                   **: *:*****:***********:*:**:***********  ***:***::*:*******

Synechococcus      TPTLISEPTGPMTVVMTAVIAHFTASAATPEEGLAIAFTVVMMAGVFQIIFGSLKLGKYV      120
RintRC3892         TPTLISEPTGPMTVIMTGVIASMV--AKDPDNGLAMGFTVVMLAGVFQILFGIFKLGRYI      118
                   **************:**.*** :.  *  *::***:.*****:******:** :***:*:

Synechococcus      TMMPYTVISGFMSGIGIILVILQLAPFLGQASPGGGVIGTLQNLPTLLSNIQPGETALAL      180
RintRC3892         TLMPYSVISGFMSGIGFILVILQIAPLLGEKAPKGGVIGTVTNLPTLLANINPPDLILGG      178
                   *:***:**********:******:**:**: :* ******: ******:**:* :  *.

Synechococcus      GTVAIIWFMPEKFKKVIPPQLVALVLGTVIAFFVFPPEVSDLRRIGEIRAGFPELVRPSF      240
RintRC3892         LTIAILFLTPSKLKRFIPPQLIALVVATLISITVFQ-NVENIARIPEIPAALPELRLPYF      237
                    *:**::: *.*:*:.*****:***:.*:*:: **  :*.:: ** ** *.:***  * *

Synechococcus      SPVEFQRMILDAAVLGMLGCIDALLTSVVADSLTRTEHNSNKELIGQGLGNLFSGLFGGI      300
RintRC3892         TPEEVTTMVVDGVVLGMLGSIDTLLTAVIADSLTRTEHKSDKELIGQGVANLISGLCGGL      297
                   :* *.  *::*..******.**:***:*:*********:*:*******:.**:*** **:

Synechococcus      AGAGATMGTVVNIQSGGRTALSGLVRAFVLLVVILGAASLTATIPLAVLAGIAFKVGVDI      360
RintRC3892         PGAGATMGTVVNIQTGARTALSGITRALVLLVVVVGAASLAQNIPMSVLAGIALKVGFDI      357
                    *************:*.******:.**:*****::*****: .**::******:***.**

Synechococcus      IDWSFLKRAHEISPKGALIMYGVILLTVLVDLIVAVGVGVFVANVLTIERMSNLQSEKVQ      420
RintRC3892         LDWSFLKRAHKVSLKGALIMYGVLLLTVFVDLIVAVGVGVFIANILTIERLSKLQSQEVK      417
                   :*********::* *********:****:************:**:*****:*:***::*:

Synechococcus      TVSDADDNIRLTTTEKRWLDEGQGRVLLFQLSGPMIFGVAKAIAREHNAMGDCDALVFDI      480
RintRC3892         TITDADDAITLTEKEKALLDEANGRVLLLYLSGPMIFGVSKAIAREHSAMRDADALILDL      477
                   *::**** * ** .**  ***.:*****: *********:*******.** *.***::*:

Synechococcus      GEVPHMGVTASLALENAIEEALDKERQVYIVGAAGQTRRRLEKLKLFKRVPPDKCLMSRE      540
RintRC3892         SDVPMLGVTACLAIENAIKDAVDKGLQVFIVGATGKVKRRLEKFGLSQFLPTDHLLVDRT      537
                   .:** :****.**:****::*:**  **:****:*:.:*****: * : :* *: *:.*

Synechococcus      EALKNAVLGIYPH---LADGVTAPSSEMG--       566
RintRC3892         EALQQAVASIKGYTPNTSNNV-APIDDSPVL       567
                   ***::** .*  :    ::.* ** .:
```

Fig. S2. Clustal W alignment (continued). (B) Alignment of BicA from *Synechococcus* (Ga0265390_10577) and RintRC_4851. **(C)** Alignment of BicA from *Synechococcus* (Ga0265390_10577) and RintHH_3990-60.

B

```
Synechococcus       MQITNKIHFRNIRGDIFGGLTAAVIALPMALAFGVAS--GAGAEAGLWGAVLVGFFAALF      58
RintRC4851          ---------------MLGGITAAIVALPLALAFGVSASRQVGAIAGLYGAIFVGLFAALF      45
                                   ::**:***::***:******::   .** ***:**::**:*****

Synechococcus       GGTPTLISEPTGPMTVVMTAVIAHFTASAATPEEGLAIAFTVVMMAGVFQIIFGSLKLGK      118
RintRC4851          GGTPSQISGPTGPMTVVMATVFSTM--IAKNPDNGIAMAFTVVILGGLFQILFGVMRLGK      103
                    ****: ** *********::*:: :   * .*::*:*:*****::.*:***:** ::***

Synechococcus       YVTMMPYTVISGFMSGIGIILVILQLAPFLGQASPGGGVIGTLQNLPTLLSNIQPGETAL      178
RintRC4851          YITFIPYTVISGFMSGIGFIIIFLQIAPLLGHPSS-AKVIDSLANLPEYLTTPNPIATGL      162
                    *:*::*************:*:::**:**:**: *  . **.:* ***  *:. :*  *.*

Synechococcus       ALGTVAIIWFMPEKFKKVIPPQLVALVLGTVIAFFVFPPEVSDLRRIGEIRAGFPELVRP      238
RintRC4851          GLLTLLIVFASPPKLNRIVPSPLIALIVGTVISVTLFPD--SNLPQIGKIPTGLPQIQLP      220
                    .* *: *::  * *:::::*  *:**::****:. :**   *:* :**:* :*:*::  *

Synechococcus       SFSPVEFQRMILDAAVLGMLGCIDALLTSVVADSLTRTEHNSNKELIGQGLGNLFSGLFG      298
RintRC4851          VFSPNQIKDMVLYALMLATLGAIDSLLTSLVADNITRTQHDSDKELIGQGIGNCIAGLFG      280
                     *** ::: *:* * :*. **.**:****:***.:***:*:*******:**   ::****

Synechococcus       GIAGAGATMGTVVNIQSGGRTALSGLVRAFVLLVVILGAASLTATIPLAVLAGIAFKVGV      358
RintRC4851          GLPGAGATMRTVVNVRAGGKTALSGVIHALVLLVIVLGAGKLTENIPNAVLAGILIKVGI      340
                    *: ****** ****:::**:*****:::*:****::***..** .** ****** :***:

Synechococcus       DIIDWSFLKRAHEISPKGALIMYGVILLTVLVDLIVAVGVGVFVANVLTIERMSNLQSEK      418
RintRC4851          DIIDWGFLKRAHRISLRGAGLMYGVLLLTVFVDLIVAVGLGVFLANLLTLKRLSDLQAEQ      400
                    *****.******.** :** :****:****:********:***:**:**::*:*:**:*:

Synechococcus       VQTVSDADDNIRLTTTEKRWLDEGQGRVLLFQLSGPMIFGVAKAIAREHNAMGDCDALVF      478
RintRC4851          IQAIVTPNEQILLSSAEKDILRQAQGRILLFHLGGPMSFGAAKSISRRLSIVEDYDVLIL      460
                    :*::   :::* *:::**  * :.***:***:*.*** **.**:*:*. . : * *.*::

Synechococcus       DIGEVPHMGVTASLALENAIEEALDKERQVYIVGAAGQTRRRLEKLKLFKRVPPDKCLMS      538
RintRC4851          DLSEVPHIGVTASLAIENMVKEACEKRRSIFLVGASGKVRERLRHLELLDRLLATNQLPS      520
                    *:.****:*******:** ::** :*.*.:::***:*:.*.**.:*:*:.*:   : * *

Synechococcus       REEALKNAVLGIYPHLADGVTAPSSEMG 566
RintRC4851          RLEALETGLVIVRERH------------ 536
                    * ***:..:: :  :
```

C

```
Synechococcus       MQITNKIHFRNIRGDIFGGLTAAVIALPMALAFGVASGAGAEAGLWGAVLVGFFAALFGG      60
RintHH3990-60       MQILNRIHFRNLRGDIFGGLTSAIISLLLAIAFSVASGMRPISGVYGAVIIGLFAALFGV      60
                    *** *:*****:*********:*:*:* :*:**.****    :*::***::*:******

Synechococcus       TPTL-ISEPTGPMTVVMTAVIAHFTASAATPEEGLAIAFTVVMMAGVFQIIFGSLKLGKY      119
RintHH3990-60       TPTLIISEPTGPMTVIMTGVIASMIAK---------------------------------      87
                    **** **********:**.*** : *.

Synechococcus       VTMMPYTVISGFMSGIGIILVILQLAPFLGQASPGGGVIGTL-QNLPTLLSNIQPGETAL      178
RintHH3990-60       ---------DTEMSGIVSILVILQIAPLVGEQVPKGGVIGTIISNIPTLITNINAPDLVL      138
                             .  ****  ******:**::*:  * ******: .*:***::**:  : .*

Synechococcus       ALGTVAIIWFMPEKFKKVIPPQLVALVLGTVIAFFVFPPEVSDLRRIGEIRAGFPELVRP      238
RintHH3990-60       GGLTITILFLTPSKLKYFFPPHLIALIIGTLVYITVL--QHPEIARIPEIPAEWPKLQLP      196
                    .  *::*::: *.*:* .:**:*:**::**:: : *:  :  :: ** ** * :*:*  *

Synechococcus       SFSPVEFQRMILDAAVLGMLGCIDALLTSVVADSLTRTEHNSNKELIGQGLGNLFSGLFG      298
RintHH3990-60       YFTPGQI---------TCYMGCIDTLLTSVIADSLTRVEHKSNKELICQDIANLISGLFG      247
                     *:* ::            :****:*****:******.**:****** *.:.**:*****

Synechococcus       GIAGAGATMGTVVNIQSGGRTALSGLVRAFVLLVVILGAASLTATIPLAVLAGIAFKVGV      358
RintHH3990-60       GLPGADATIGTIVNIQTGAKTALSGVTRSLVLIIVILSAARLAQNIPMSVLTGIALKVGL      307
                    *: **.**:**:****:*.:*****:.*::**::***.** *: .**::**:***:***:

Synechococcus       DIIDWSFLKRAHEISPKGALIMYGVILLTVLVDLIVAVGVGVFVANVLTIERMSNLQSEK      418
RintHH3990-60       DILDWNFLKCAHKVSLKGALIMYDVLFLTIFVDLIVAVGVGLFIANILTIEHLFNLQSKE      367
                    **:**.*** **::* *******.*::**::**********:*:**:****:: ****::

Synechococcus       VQTVSDADDNIRLTTTEKRWLDEGQGRVLLFQLSGPMIFGVAKAIAREHNAMGDCDALVF      478
RintHH3990-60       VKTISDTDEKINLTNIERSLLEQVNGRILLFYLNSPMIFRVAKAISREHSAMRDADALII      427
                    *:*:**:*::*.**. *:  *:: :**:*** *..**** *****:***.** *.***::

Synechococcus       DIGEVPH---MGVTASLALENAIEEALDKERQVYIVGAAGQTRRRLEKLKLFKRVPPDKC      535
RintHH3990-60       DLSDVPYQCMLGVTACLAIENTVKDGVHRGLQVFIVSAAGKVKQRLERFDLSQILPPNHL      487
                    *:.:**:   :****.**:**::::.:.:  **:**.***:.::***::.* : :**::

Synechococcus       LMSREEALKNAVLGIYPHLADGVTAPSSEMG------- 566
RintHH3990-60       LANRTEALQQALFFVIKYS--GYTS-SNILALDDSTIL 522
                    * .* ***::*:: :  :   * *: *. :.
```

Fig. S2. Clustal W alignment (continued). (D) Alignment of *Anabaena alr1633* and RintRC_3409. (E) Alignment of *Anabaena alr1633* and RintHH_20770.

D

```
Alr1633          ------------------MAGAVVGLALIPEAIAFSIIAGVDPKVGLYASFIIAVMTAFL 42
RintRC3409       MNIQKMKREWFSNVRADVLAGAVVGLALIPEAIAFSIIAGVDPKVGLYASFIIAVLTAFL 60
                                   :************************************:****

Alr1633          GGRPGSISAATGAMALLMIDLVKDHGLQYLFAATLLTGVFQVVFGFLQLGRQMRFVPRAV 102
RintRC3409       GGTPGSISAATGAMALLMIDLVKDHGLQYLLATTFLTGIIQVLFGVFKLGRQMKYVPRAV 120
                 ** ***************************:*:*:***::**:**.::*****::*****

Alr1633          MIGYINALAVLIFLAQLPQLTNVPPTVYILTLLSLGIIYILPRFTKAVPSPLVALAVMTI 162
RintRC3409       MLGYINALAVLIFMAQLPQLLGKPPIVYILTAISLAIIYILPRFTKVVPSPLVTLAVMTL 180
                 *:***********:****** . ** ***** :**.**********.******:*****:

Alr1633          AAIALKLKVPRVGDMGELPTALPSFALPQVPLTLETFKIILPYSLTLAIVGLLASFLTAS 222
RintRC3409       AAIALKLEVPTVGDMGALPTVPPVFALPQVPFTWETLQIIFPYALTLAIVGLLASFLTSS 240
                 *******:** ***** ***. * *******:* **::**:**:**************:*

Alr1633          LVDELTDTPSDKNQEAKGQGIANIVTAFFGGMAGCGMIGQSVINVQSGGRGRLSTLAAGV 282
RintRC3409       LVDELTDTPSDKNQEAKGQGIANIVTAFFGGMAGCGMIGQSVINVQSGGRGRLSTLSAGI 300
                 ********************************************************:**:

Alr1633          FLLIAILFLQDWVKQMPMAALVAVMIMVSIGTFRWTSFKNISRIPRTETAVMLTTMFVTI 342
RintRC3409       FLLFAILVLQDWVKQMPMAALVAIMIMVSIGTFSWSSFKDIRRIPPSETLVMLATMFVTI 360
                 ***:***.***************:********* *:***:* *** :** ***:******

Alr1633          FTRNFALGVVTGIVMSTVFFSNKIAQLVFVDKVLSEDGTHRIYKVVGQIFFLSRDEFLGF 402
RintRC3409       FTRNFALGVATGIIMSTVFFSRKIAKLVFVERILSEDGSHRTYIVSGQIFFLSKEEFIDS 420
                 *********.***:*******.***:****:::*****:** * * *******::**:. 

Alr1633          FDFTELLERVTIDLTHAHLWDQGAVEVLDRAVLKFRRNGAEVELIGLNQASATLLNKLAT 462
RintRC3409       FDFGEVVDTVTIDLTNAHLWDQGAVGILDRIVLKFRRNGADVELLGLNEASATLVEKLGV 480
                 *** *::: ******:********* :*** *********:***:***:*****::**..

Alr1633          HQKSDAVNKQ   472
RintRC3409       HNQTESLRK-   489
                 *::::::.* 
```

E

```
Alr1633          ------------------MAGAVVGLALIPEAIAFSIIAGVDPKVGLYASFIIAVMTAFL          42
RintHH20770      MNIQKLKREWFSNARADILAGAVVGVALIPEAIAFSIIAGVDPKVGLYASFIIAIITAFL          60
                                   :******:****************************::****

Alr1633          GGRPGSISAATGAMALLMIDLVKDHGLQYLFAATLLTGVFQVVFGFLQLGRQMRFVPRAV          102
RintHH20770      GGRPGSISAATGAMALLMIDLVKEYGLQYLLATTFLTGAIQVIFGIFKLGRQMKYVPRAV          120
                 ***********************::*****:*:*:***.:**:**:::*****::*****

Alr1633          MIGYINALAVLIFLAQLPQLTNVPPTVYILTLLSLGIIYILPRFTKAVPSPLVALAVMTI          162
RintHH20770      MLGYINALAVLIFMAQLPQLISKSPTVYILTVISLAIIYILPRFTKAVPSPLVTLAVVTS          180
                 *:***********:****** .  *******::**.*****************:***:* 

Alr1633          AAIALKLKVPRVGDMGELPTALPSFALPQVPLTLETFKIILPYSLTLAIVGLLASFLTAS          222
RintHH20770      VATTLKLEVPIVGDMGELPKTLPVFTLPEVPLSWETLQVILPYSLTLATVGLLASFLTSS          240
                 .* :***:** ********.:** *:**:***: **:::********* *********:*

Alr1633          LVDELTDTPSDKNQEAKGQGIANIVTAFFGGMAGCGMIGQSVINVQSGGRGRLSTLAAGV          282
RintHH20770      LVDELTDTPSDKNQEAKGQGIANMVTAFFGGMAGCGMIGQSVINVQSGGRGRLSTFCAGM          300
                 ***********************:*******************************:.**:

Alr1633          FLLIAILFLQDWVKQMPMAALVAVMIMVSIGTFRWTSFKNISRIPRTETAVMLTTMFVTI          342
RintHH20770      FLLFAILVLQDWVKQIPMAALVAIMIMVSIGTFRWSSFQNINVIPRSETLVMITTMVVTI          360
                 ***:***.*******:*******:***********:**:**. ***:** **:***.***

Alr1633          FTRNFALGVVTGIVMSTVFFSNKIAQLVFVDKVLSEDGTHRIYKVVGQIFFLSRDEFLGF          402
RintHH20770      LTRNFALGVVTGIIMSTVFFSRKIAKLVFVERVLSQDGSHCTYLVSGQIFFLSKEEFLES          420
                 :************:*******.***:****::***:**:*  * * *******::***  

Alr1633          FDFTELLERVTIDLTHAHLWDQGAVEVLDRAVLKFRRNGAEVELIGLNQASATLLNKLAT          462
RintHH20770      FDFGEILDSVTIDLNNAHLWDQGAVATLDRIILKFRRNGSDVKLLGLNEASATLVDKLGT          480
                 *** *:*: *****.:********* .*** :*******::*:*:***:*****::**.*

Alr1633          HQKSDAVNKQ  472
RintHH20770      HNHTESWKE-  489
                 *::::: :: 
```

Fig. S3. Amino acids in the bicarbonate binding site of BicA. Relevant fragments of the BicA proteins of *Synechocystis* sp. PCC 6803, *Synechococcus* sp. PCC 7002 and the SulP-like proteins from *Richelia* spp. are shown. The amino acid residues involved in bicarbonate binding in the BicA protein of *Synechocystis* sp. PCC 6803 are indicated in red color. The six amino acids involved in binding of bicarbonate are conserved in the proteins from PCC 6803, PCC 7002, RintRC_3892 and RintRC_4851, but not in all the SulP-like proteins. Conservation is indicated in red shade or, to highlight full conservation, in blue shade. The following symbols indicate residue conservation: * = identical residues; : = strong similarity; . = weak similarity; (blank) = no conservation.

```
                                           T69
RintRC_3409                   AFLGGTPGSISAATGAMALLMIDLV---------KDHGLQYLLATTFLTGIIQVLFGVFK   108
RintHH_20770                  AFLGGRPGSISAATGAMALLMIDLV---------KEYGLQYLLATTFLTGAIQVIFGIFK   108
RintHH3990-60                 ------------------------------------------------------------   0
RintRC_4851                   ALFGGTPSQISGPTGPMTVVMATVFSTMI--AKNPDNGIAMAFTVVILGGLFQILFGVMR   118
RintRC_3892                   ALFGGTPTLISEPTGPMTVIMTGVIASMV--AKDPDNGLAMGFTVVMLAGVFQILFGIFK   113
BicA_Synechocystis_PCC6803    ALFGGTPTLISEPTGPMTVVQTAVIASLV--AADPDNGLAMAFTVVMMAGLFQIAFGLLK   113
BicA_Synechococcus_PCC7002    ALFGGTPTLISEPTGPMTVVMTAVIAHFTASAATPEEGLAIAFTVVMMAGVFQIIFGSLK   115

RintRC_3409                   LGRQMKYVPRAVMLGYINALAVLIFMAQLPQLLGKPPI---------------------V   147
RintHH_20770                  LGRQMKYVPRAVMLGYINALAVLIFMAQLPQLISKSPT---------------------V   147
RintHH3990-60                 ----------------MLGVTACLAI--------ENTVKDGVH-----------------   19
RintRC_4851                   LGKYITFIPYTVISGFMSGIGFIIIFLQIAPLLGHPSS-AKVIDSLANLPEYLTTPNPIA   177
RintRC_3892                   LGRYITLMPYSVISGFMSGIGFILVILQIAPLLGEKAPKGGVIGTVTNLPTLLANINPPD   173
BicA_Synechocystis_PCC6803    LGKYVTMMPYTVISGFMSGIGIILVILQLAPFLGQASPKGGVIGTLQALPNLVSNVRPVE   173
BicA_Synechococcus_PCC7002    LGKYVTMMPYTVISGFMSGIGIILVILQLAPFLGQASPGGGVIGTLQNLPTLLSNIQPGE   175
                                              : .:   : :        .

RintRC_3409                   YILTAISLAII-YILPRFTKVVPSPLVTLAVMTLAAIALKL-EVPTVGDMGALPTV-PPV   204
RintHH_20770                  YILTVISLAII-YILPRFTKAVPSPLVTLAVVTSVATTLKL-EVPIVGDMGELPKT-LPV   204
RintHH3990-60                 RGLQVFIVSAAGKVKQRLERFDLSQI-------------LPPNHLLANRTEALQQALFFV   66
RintRC_4851                   TGLGLLTLLIVFASPPKLNRIVPSPLIALIVGTVISVTLFP--DSNLPQIGKIPTG-LPQ   234
RintRC_3892                   LILGGLTIAILFLTPSKLKRFIPPQLIALVVATLISITVFQN-VENIARIPEIPAA-LPE   231
BicA_Synechocystis_PCC6803    TLLALMTVGIIWFMPSRWKKFAPPQLVALVLGTIISITLFG--DLDIRRIGEIQAG-LPA   230
BicA_Synechococcus_PCC7002    TALALGTVAIIWFMPEKFKKVIPPQLVALVLGTVIAFFVFPPEVSDLRRIGEIRAG-FPE   234
                                *    :        :  :     :                          :
                                                          D258 T262
RintRC_3409                   FALPQVPFTWETLQIIFPYALTLAIVGLLASFLTSSLVDELTDTPSDKNQEAKGQGIANI   264
RintHH_20770                  FTLPEVPLSWETLQVILPYSLTLATVGLLASFLTSSLVDELTDTPSDKNQEAKGQGIANM   264
RintHH3990-60                 IKYS--GYT---SSNILALDDSTILMGCIDTLLTSVIADSLTRVEHKSNKELICQDIANL   121
RintRC_4851                   IQLP--VFSPNQIKDMVLYALMLATLGAIDSLLTSLVADNITRTQHDSDKELIGQGIGNC   292
RintRC_3892                   LRLP--YFTPEEVTTMVVDGVVLGMLGSIDTLLTAVIADSLTRTEHKSDKELIGQGVANL   289
BicA_Synechocystis_PCC6803    LQLP--VFQADQLQRMLIDAAVLGMLGCIDALLTSVVADSLTRTEHNSNKELVGQGIGNV   288
BicA_Synechococcus_PCC7002    LVRP--SFSPVEFQRMILDAAVLGMLGCIDALLTSVVADSLTRTEHNSNKELIGQGLGNL   292
                              :              :.        :* : ::**: :.*.:* .  ..::*   *.:.*

RintRC_3409                   VTAFFGGMAGCGMIGQSVINVQSGGRGRLSTLSAGIFLLFAILVLQDWVKQMPMAALVAI   324
RintHH_20770                  VTAFFGGMAGCGMIGQSVINVQSGGRGRLSTFCAGMFLLFAILVLQDWVKQIPMAALVAI   324
RintHH3990-60                 ISGLFGGLPGADATIGTIVNIQTGAKTALSGVTRSLVLIIVILSAARLAQNIPMSVLTGI   181
RintRC_4851                   IAGLFGGLPGAGATMRTVVNVRAGGKTALSGVIHALVLLVIVLGAGKLTENIPNAVLAGI   352
RintRC_3892                   ISGLCGGLPGAGATMGTVVNIQTGARTALSGITRALVLLVVVVGAASLAQNIPMSVLAGI   349
BicA_Synechocystis_PCC6803    MSGLFGGLGGAGATMGTVVNIQSGGRTALSGLIRAMVLLVVILGAAKLAATIPLAVLAGI   348
BicA_Synechococcus_PCC7002    FSGLFGGIAGAGATMGTVVNIQSGGRTALSGLVRAFVLLVVILGAASLTATIPLAVLAGI   352
                              .:.: **: *..    :::*:::*.:  ** .  .:.*:. ::     .  :* :.*..*
                                         G300
                                         A301
                                         T302
```

Fig. S4. Cloning strategy to express *Richelia* genes in the *Synechocystis* △5 mutant. The restriction sites used for cloning were: NdeI and SphI for RintRC_3892, NdeI and XhoI for RintRC_4851, BamHI and BamHI for RintRC_3409, RintHH_3990-60 and RintHH_20770. The RintRC_3892 gene was PCR-amplified and cloned in pSpark prior to transfer to the NdeI/SphI site of pNRSD_PcpcB_Ery.

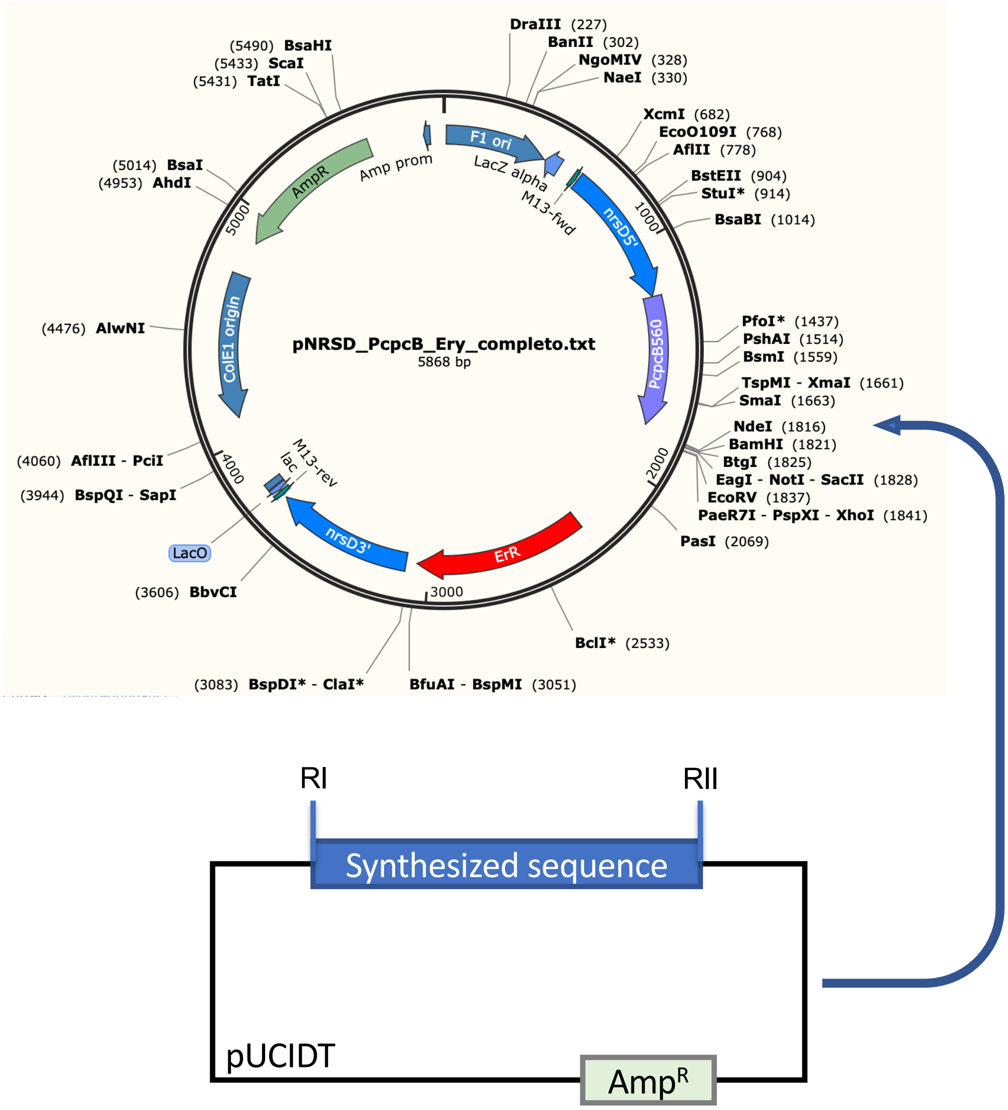

Fig. S5. DNA sequence covering the RintHH_3960-3970-3980-3990 *sulP*-like gene fragments. The RintHH_3960-3970-3980-3990 genes (green, blue, magenta and yellow letters, respectively) were cloned together with their natural intervening DNA sequences (*upper panel*). The transcript from the cloned DNA fragment should be translated producing four polypeptides that align with consecutive fragments of BicA from *Synechococcus* sp. PCC 7002 (*lower panel*).

```
GGATCCATGCAAATTTTGAATCGCATCCATTTTAGAAATCTTCGTGGTGACATCTTTGGAGGTTTAACCTCAG
CGATTATCTCCTTGCTCTTAGCTATTGCATTCAGTGTTGCTTCTGGGATGAGACCAATATCAGGTGTATATGG
TGCTGTGATAATAGGTTTATTTGCAGCATTATTTGGTGTTACACCAACCCTAATAATTTCCGAACCCACTGGGC
CAATGACTGTCATCATGACTGGTGTTATAGCTTCTATGATAGCAAAGGATACAGAGTAGCGTGGCAATGGCAT
TCACGGTGGTAATCTTAGCAGGTATGATTTAATTACTGTTTGGTATATTTAAACCAGGCAAATATATTACCTTAA
TGCCTTATAGTGTTATTTCTGGCTTCATGTCGGGAATTGTTTCAATTCTAGTGATTTTACAAATTGCCCCATTG
GTGGGCGAACAAGTACCAAAAGGTGGGGTAATTGGCACTATTATTAGCAATATTCCAACCTTAATTACTAATA
TTAATGCCCCTGATTTGGTATTAGGAGGGTTAACAATCACTATTTTATTCCTAACACCATCAAAATTGAAGTATT
TCTTTCCTCCACATTTAATTGCATTAATTATTGGTACTCTAGTTTATATAACTGTTTTGCAACATCCTGAGATAG
CAAGAATACCAGAAATCCCTGCAGAATGGCCAAAACTACAATTACCGTATTTCACACCCGGGCAAATAACTTG
CTATTAGATAGTATTGTTTTGGCAATAATGGGGTGTATTGATACTCTTTTAACCTCTGTAATTGCAGATAGTTTA
ACCCGTGTTGAGCATAAATCTAATAAAGAACTAATTTGTCAGGACATTGCTAACTTAATTTCTGGCTTGTTTGG
GGGTCTACCTGGTGCAGATGCAACTATAGGAACTATAGTTAACATTCAAACAGGAGCTAAAACTGCACTATCA
GGAGTAACTCGTAGTTTAGTATTAATAATAGTAATTTTGAGTGCTGCAAGACTAGCTCAAAATATTCCCATGTC
TGTATTGACTGGTATTGCCTTAAAAGTGGGTCTTGATATCCTAGATTGGAATTTCCTCAAGTGTGCTCACAAA
GTATCACTTAAGGGTGCGCTAATTATGTATGATGTATTATTTTTAACAATATTTGTAGATTTAATTGTTGCTGTA
GGTGTAGGTTTATTCATAGCAAATATTTTGACTATTGAGCATCTTTTTAACCTTCAATCAAAAGAAGTTAAAACT
ATTAGTGATACTGATGAAAAAATTAATCTAACTAATATAGAGAGGTCTTTACTTGAACAAGTAAATGGAAGAAT
ACTGTTATTTTACCTTAATAGTCCTATGATATTTAGGGTGGCAAAAGCAATTTCTCGAGAGCATTCAGCGATGA
GAGATGCAGATGCTCTCATAATAGATTTAAGTGATGTACCGTACCAATGTTAGGAGTAACTGCTTGTTTGGCA
ATTGAAAACACAGTTAAAGATGGAGTTCATAGAGGTCTGCAAGTATTTATTGTGAGTGCTGCAGGTAAGGTTA
AACAGCGTTTAGAACGATTTGACTTATCACAAATTTTACCTCCCAACCACTTGTTGGCAAATCGTACAGAGGC
ACTTCAGCAAGCACTTTTTTTTGTAATAAAATACTCAGGCTACACAAGTAGTAACATTCTAGCATTGGATGATT
CAACCATTTTGTAGGGATCC
```

```
Synechococcus      MQITNKIHFRNIRGDIFGGLTAAVIALPMALAFGVASGAGAEAGLWGAVLVGFFAALFGG 60
RintHH3990-60      MQILNRIHFRNLRGDIFGGLTSAIISLLLAIAFSVASGMRPISGVYGAVIIGLFAALFGV 60
                   *** *:*****:*********:*:*:* :*:**.****     :*::***::*:******

Synechococcus      TPTL-ISEPTGPMTVVMTAVIAHFTASAATPEEGLAIAFTVVMMAGVFQIIFGSLKLGKY 119
RintHH3990-60      TPTLIISEPTGPMTVIMTGVIASMIAK--------------------------------- 87
                   **** **********:**.*** : *.

Synechococcus      VTMMPYTVISGFMSGIGIILVILQLAPFLGQASPGGGVIGTL-QNLPTLLSNIQPGETAL 178
RintHH3990-60      ---------DTEMSGIVSILVILQIAPLVGEQVPKGGVIGTIISNIPTLITNINAPDLVL 138
                            .  ****  ******:**::*:  * ******: .*:***::**:  : .*

Synechococcus      ALGTVAIIWFMPEKFKKVIPPQLVALVLGTVIAFFVFPPEVSDLRRIGEIRAGFPELVRP 238
RintHH3990-60      GGLTITILFLTPSKLKYFFPPHLIALIIGTLVYITVL--QHPEIARIPEIPAEWPKLQLP 196
                   .  *::*::: *.*:* .:**:*:**::**:: : *:   :  :: ** ** * :*:*  *

Synechococcus      SFSPVEFQRMILDAAVLGMLGCIDALLTSVVADSLTRTEHNSNKELIGQGLGNLFSGLFG 298
RintHH3990-60      YFTPGQI---------TCYMGCIDTLLTSVIADSLTRVEHKSNKELICQDIANLISGLFG 247
                    *:* ::              :****:*****:******.**:****** *.:.**:*****

Synechococcus      GIAGAGATMGTVVNIQSGGRTALSGLVRAFVLLVVILGAASLTATIPLAVLAGIAFKVGV 358
RintHH3990-60      GLPGADATIGTIVNIQTGAKTALSGVTRSLVLIIVILSAARLAQNIPMSVLTGIALKVGL 307
                   *: **.**:**:****:*.:*****:.*::**::***.** *: .**::**:***:***:

Synechococcus      DIIDWSFLKRAHEISPKGALIMYGVILLTVLVDLIVAVGVGVFVANVLTIERMSNLQSEK 418
RintHH3990-60      DILDWNFLKCAHKVSLKGALIMYDVLFLTIFVDLIVAVGVGLFIANILTIEHLFNLQSKE 367
                   **:**.*** **::* *******.*::**::**********:*:**:****:: ****::

Synechococcus      VQTVSDADDNIRLTTTEKRWLDEGQGRVLLFQLSGPMIFGVAKAIAREHNAMGDCDALVF 478
RintHH3990-60      VKTISDTDEKINLTNIERSLLEQVNGRILLFYLNSPMIFRVAKAISREHSAMRDADALII 427
                   *:*:**:*::*.**. *:  *:: :**:*** *..**** *****:***.** *.***::

Synechococcus      DIGEVPH---MGVTASLALENAIEEALDKERQVYIVGAAGQTRRRLEKLKLFKRVPPDKC 535
RintHH3990-60      DLSDVPYQCMLGVTACLAIENTVKDGVHRGLQVFIVSAAGKVKQRLERFDLSQILPPNHL 487
                   *:.:**:   :****.**:**::::.:.:  **:**.***:.::***::.* : :**::

Synechococcus      LMSREEALKNAVLGIYPHLADGVTAPSSEMG-------       566
RintHH3990-60      LANRTEALQQALFFVIKYS--GYTS-SNILALDDSTIL       522
                   * .* ***::*:: :  :   * *: *. :.
```

Fig. S6. PCR analysis showing the presence of the *Richelia* constructs in the *Synechocystis* Δ5 mutant transformants. PCR analysis was performed with the primers indicated in **(A)** and DNA isolated from the *Synechocystis* strain indicated in **(B, C)**: WT, the Δ5 mutant or the Δ5 mutant transformed with the indicated gene and grown as described in Materials and Methods. Note that, in C, three different clones of Δ5 + RintRC_3892 are shown. X symbols indicate approximate sites of recombination with the *Synechocystis* chromosome. RI and RII, restriction endonuclease cutting sites (see Fig. S3). 1, 1-kb DNA ladder (Biotools); 2-8 or 2-7, DNA from the strains indicated next to the gel.

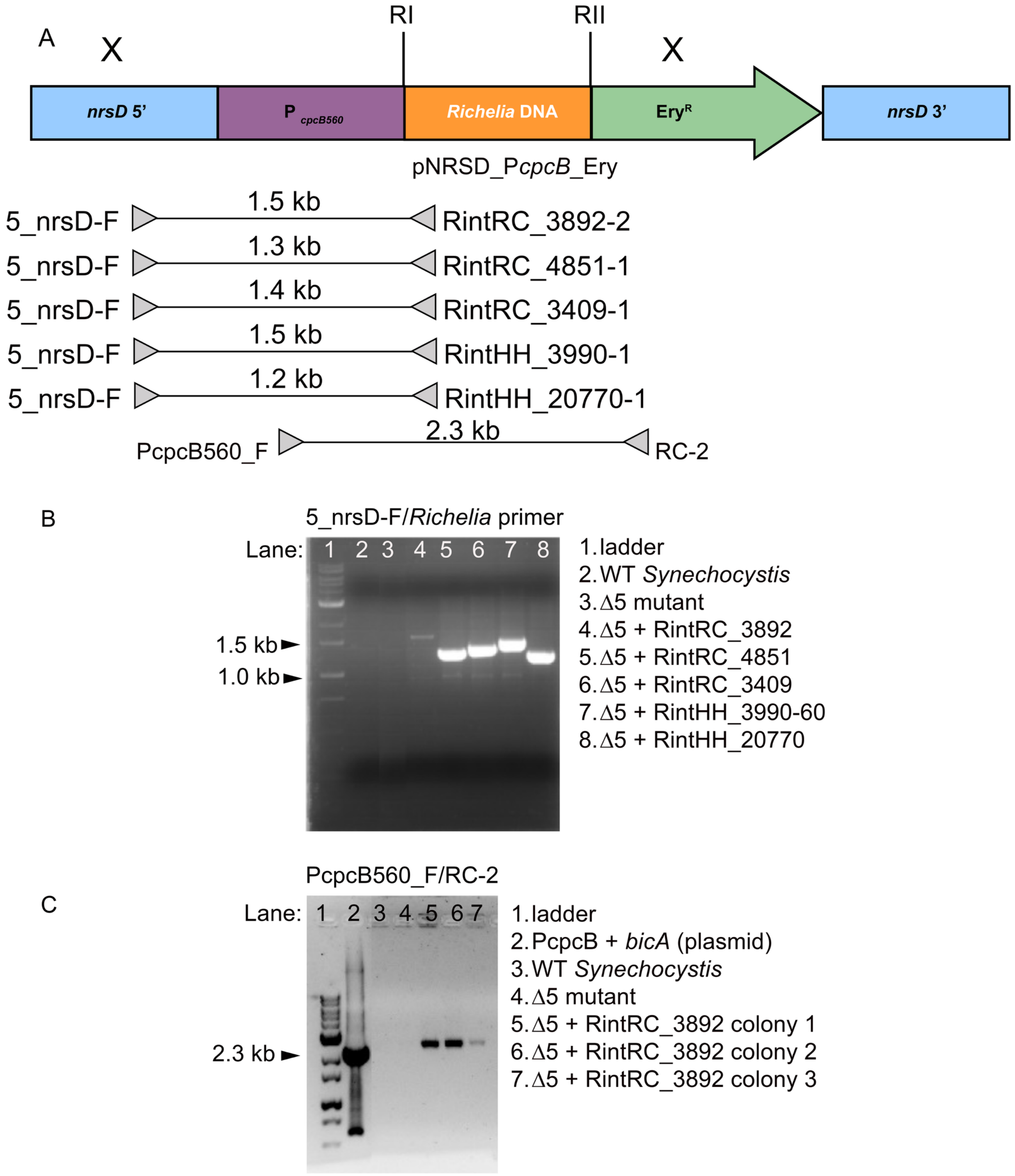

Fig. S7. Summary of qPCR analysis of expression of *bicA*. RNA was isolated from *Synechocystis* WT, the Δ5 mutant, and the Δ5 mutant complemented with RintRC_3892, RintRC_4851, RintRC_3409, RintHH_20770, RintHH_3990-60. **(A)** qPCR analysis was performed with primers specific for *Synechocystis bicA* (*Synechocystis* WT, Δ5 mutant), and the various transformants; and for normalization, *secA of Synechocystis* WT. *Synechocystis* WT [*3892*] shows the amplification from *Synechocystis* WT RNA using RintRC_3892 primers, which was background amplification as expected. The values are mean normalized expression (average ± standard deviation, SD) from three separate replicate cultures of each cell line. Note that the *Synechocystis bicA* primers amplify RNA from *Synechocystis* WT and a *bicA* fragment remaining in the Δ5 mutant because this mutant is a gentamicin-resistance cassette insertion mutant; *bicA* transcripts are detected at higher levels in the Δ5 mutant than in the WT because many of those transcripts are produced from the promoter of the gentamicin-resistance cassette. **(B)** Scheme of *bicA* (*sll0834*) inactivation strategy in the Δ5 mutant and location of primers used for qPCR. Gentamycin-resistant cassette was used to inactivate *bicA* (20).

A

**RNA (cell line)**	**Primers to amplify:**	**Normalized cDNA (average ± SD)**
Synechocystis WT	WT *bicA*	6.73 ± 8.53
Δ5 mutant	WT *bicA*	18.49 ± 4.99
Synechocystis WT	RintRC_3892	$2.29 \times 10^{-3} \pm 2.29 \times 10^{-4}$
Δ5 + RintRC_3892	RintRC_3892	68.9 ± 30.3
Δ5 + RintRC_4851	RintRC_4851	1.18 ± 0.35
Δ5 + RintRC_3409	RintRC_3409	2.05 ± 0.026
Δ5 + RintHH_20770	RintHH_20770	2.09 ± 1.10
Δ5 + RintHH_3990_60	RintHH_3990_60	5.65 ± 1.06

B

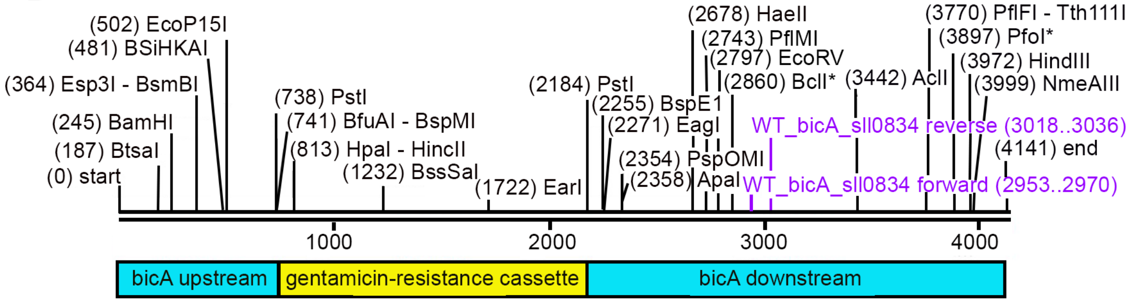

Fig. S8. Growth of WT *Synechocystis* and the transformed Δ5 mutant in air levels of CO_2 supplemented or not with bicarbonate. Photoautotrophic growth at 30 ºC under the indicated conditions: **(A)** bubbled cultures, (**B**) shaken cultures. 1, *Synechocystis* WT; 2, Δ5; 3, +RintHH_3990-60; 4, +RintHH_20770; 5, +RintRC_3409; 6, +RintRC_4851; 7, +RintRC_3892.

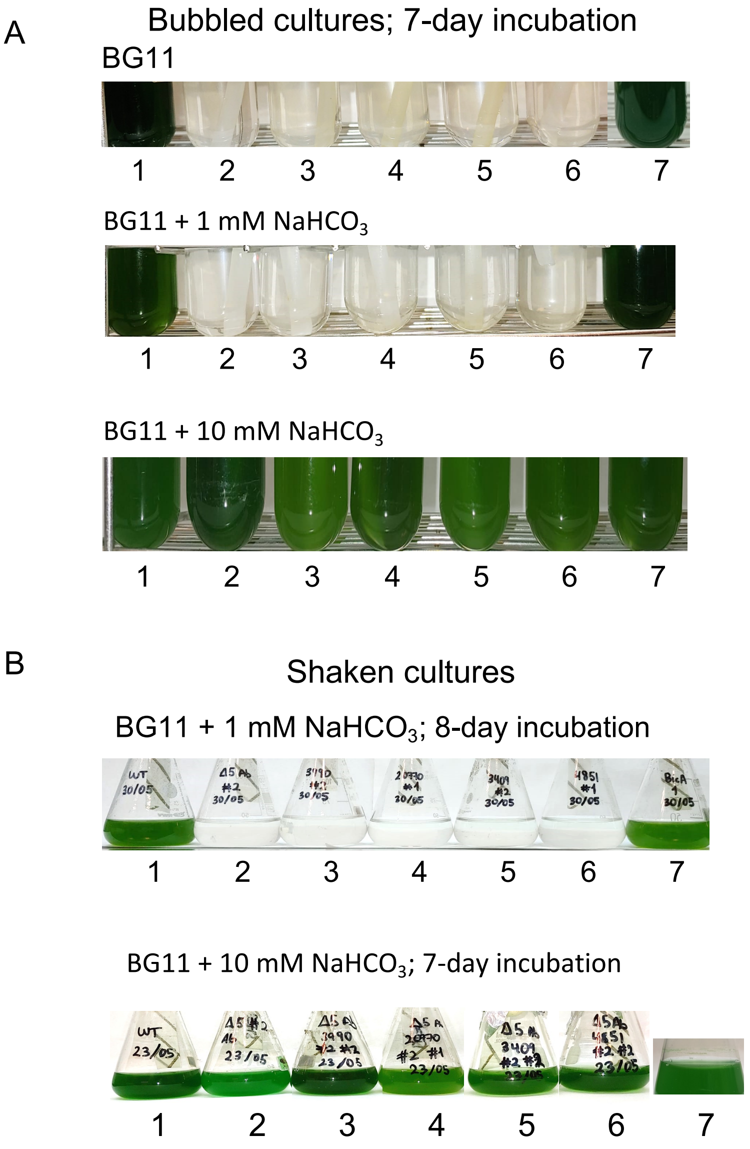

Fig. S9. Uptake of ^{14}C-bicarbonate at pH 6, pH 7 and pH 9.3 by WT *Synechocystis* and the Δ5 mutant transformed with the indicated constructs. Uptake was tested with 1 mM $NaH^{14}CO_3$ in BG11/2 medium with 25 mM MES-KOH (pH 6), TES-KOH (pH 7) or Bis-Tris-Propane-HCl (pH 9.3) buffer; final Na^+ concentration, about 33 mM. Assays were 1 min in duration. For the pH 9.3 experiment, the mean and standard deviation of three independent cultures with similar results are shown.

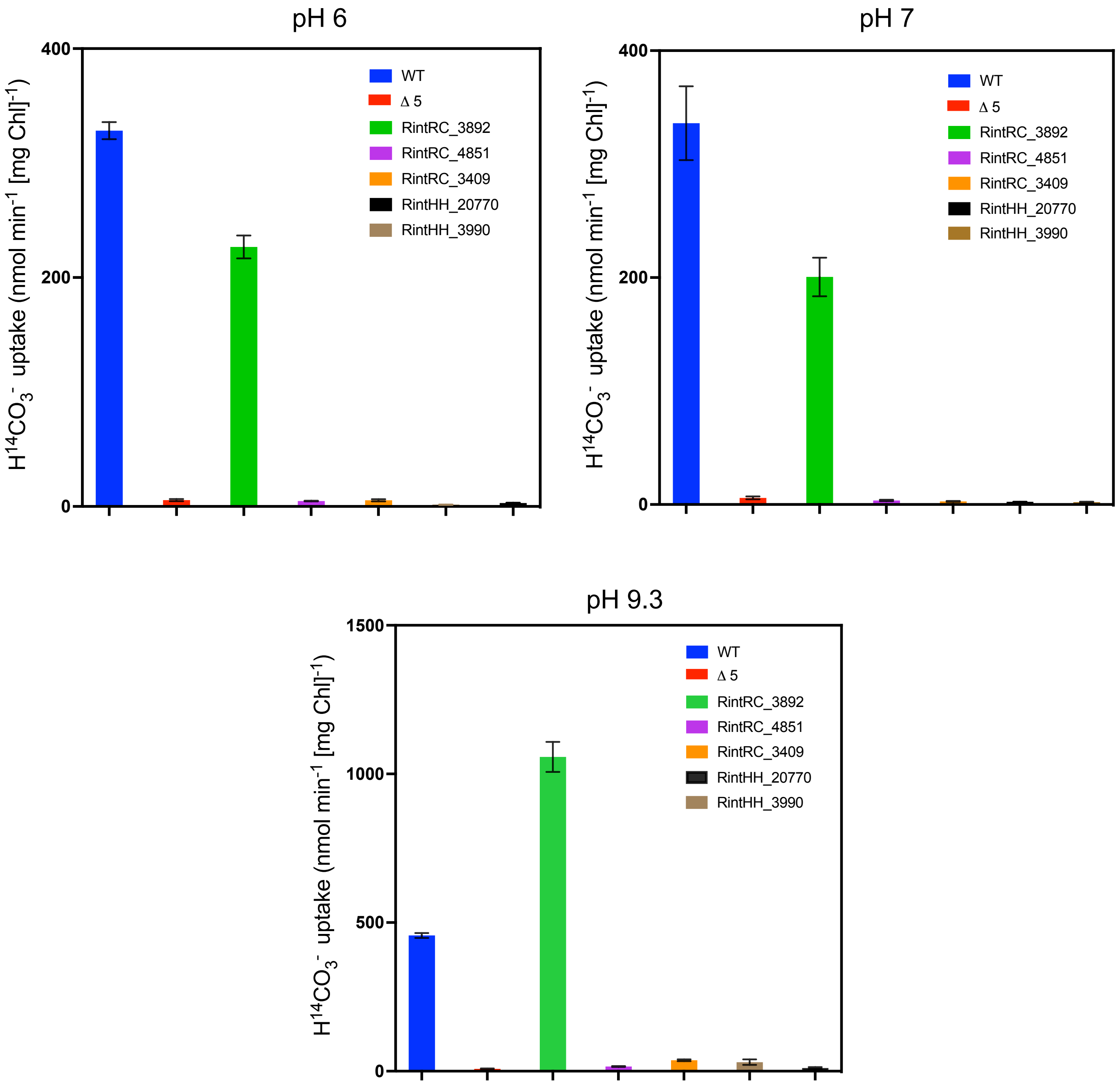

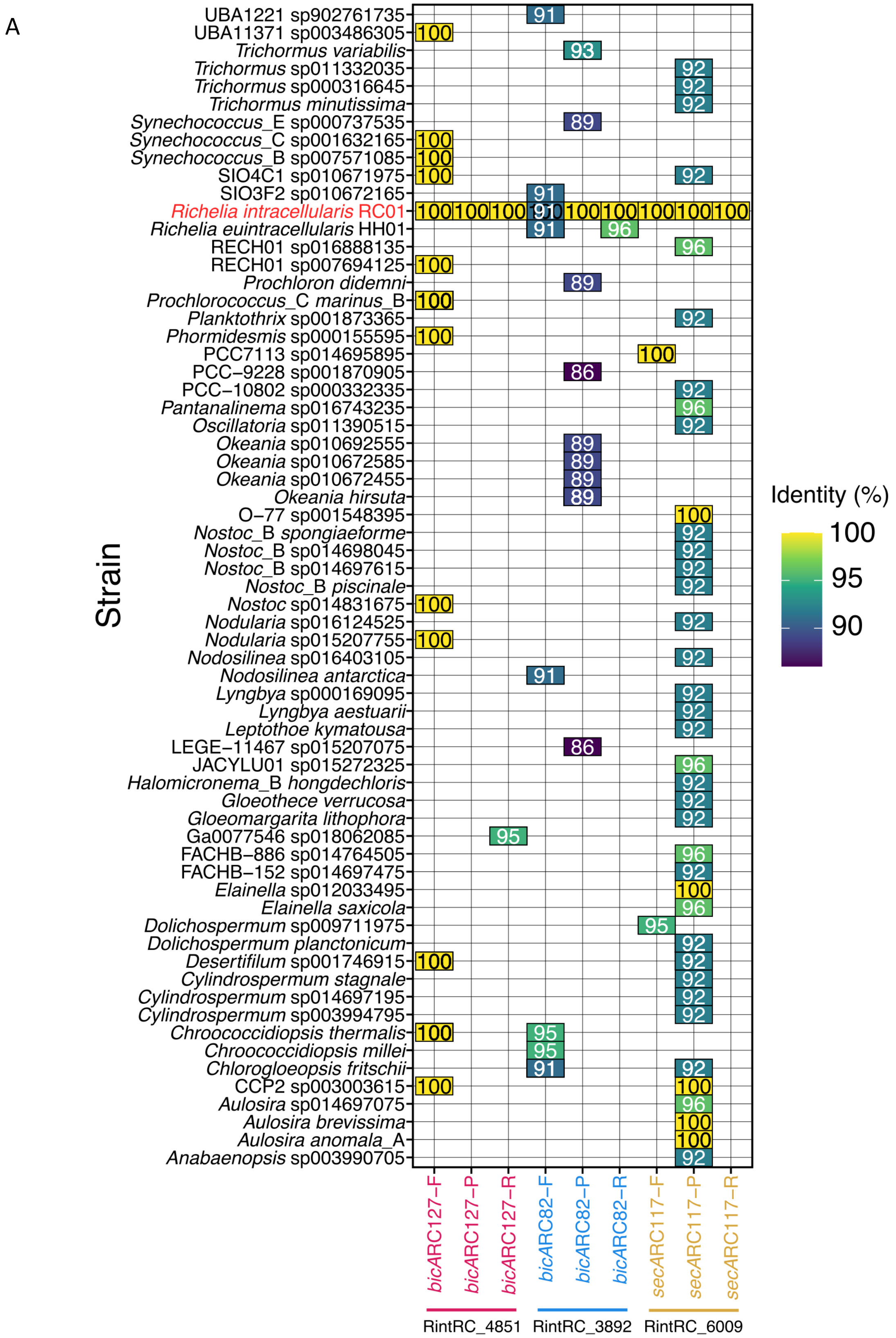

Fig. S10. **Summary of the specificity testing of the oligonucleotides used in the RT-qPCR assays for estimating the expression of the SulP-like transporters in field samples by BLASTn analyses (details of databases in Supp. Methods).** Two oligonucleotide sets were designed based on RintRC_3892 and RintRC_4891; an additional oligonucleotide set was developed for normalizing expression based on *secA* (RintRC_6009) (Suppl. Table S2). **(A)** Results of BlastN analyses using a local database containing genomes of cyanobacteria originating from RefSeq and GenBank (9,680,513 sequences) based on phylogenetic classification according to the Genome Taxonomy Database (GTDB version R207). Only alignments that used the whole query sequence were used in the analyses. The x-axis indicates the primer used as a query (bicARC127, RintRC_4851; bicARC87, RintRC_3892; secARC117, RintRC_6009). The primer names are in the structure of 'gene name', 'length of amplified sequence' and forward (F), reverse (R) or probe (P).

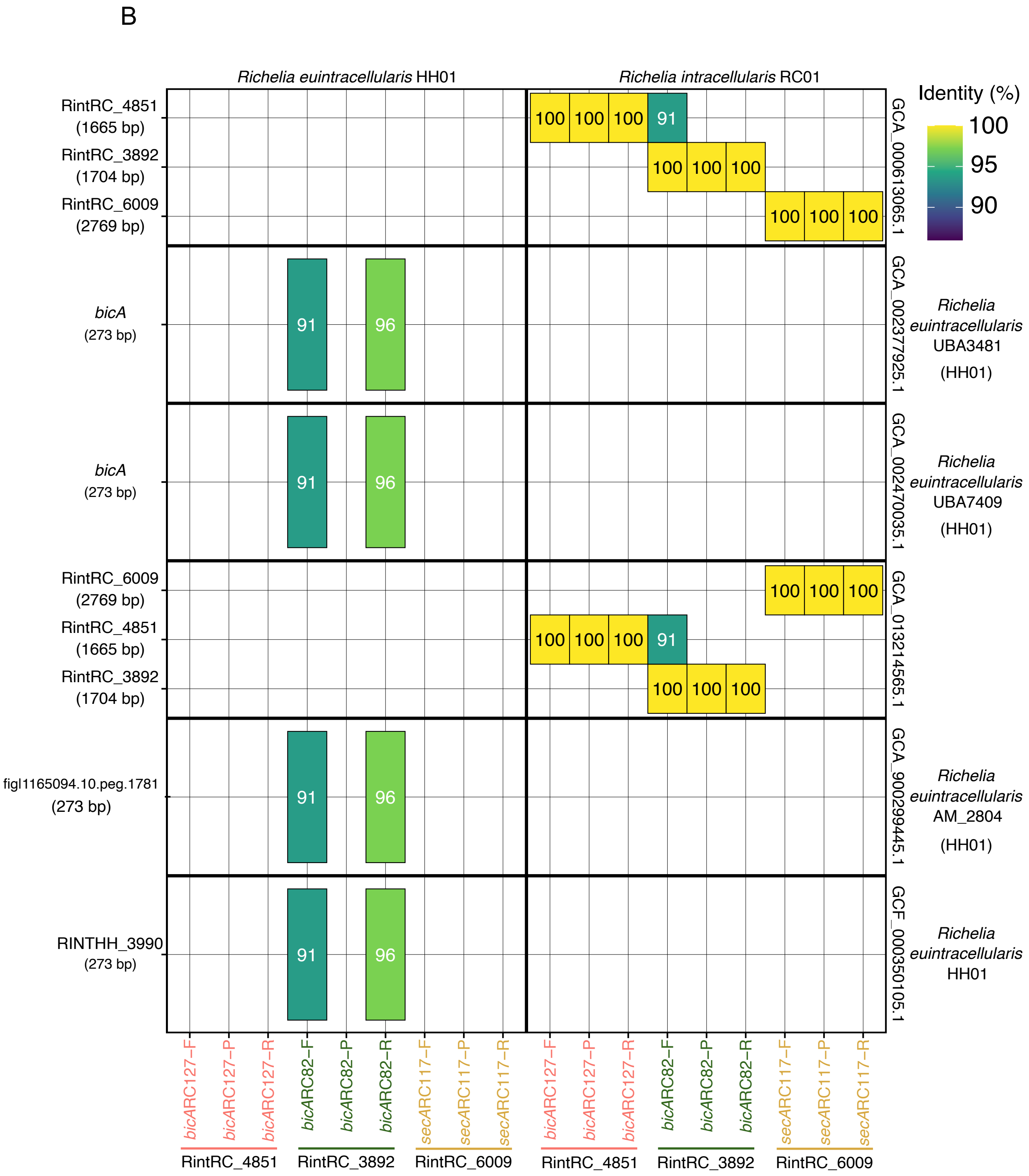

Fig. S10 (continued). **(B)** Results of BlastN analyses from Fig S7A were filtered for *Richelia* strains, which also included environmental *Richelia* MAGs.

C

```
>JHFCPCOE_05849 Bicarbonate transporter BicA [GCA_000613065.1
d__Bacteria;p__Cyanobacteria;c__Cyanobacteriia;o__Cyanobacteriales;f__Nostocaceae;g__Richelia;s__Richelia
intracellularis_B]
Length=1665

 Score = 31.9 bits (34),  Expect = 6.0
 Identities = 20/22 (91%), Gaps = 0/22 (0%)
 Strand=Plus/Plus

Query  1    GGTTTATTTGCAGCGTTGTTTG  22
            |||||||||||||| || ||||
Sbjct  169  GGTTTATTTGCAGCTTTATTTG  190
>IPCPKDNE_01849 Bicarbonate transporter BicA [GCF_000350105.1
d__Bacteria;p__Cyanobacteria;c__Cyanobacteriia;o__Cyanobacteriales;f__Nostocaceae;g__Richelia;s__Richelia
intracellularis]
Length=273

 Score = 31.9 bits (34),  Expect = 6.0
 Identities = 20/22 (91%), Gaps = 0/22 (0%)
 Strand=Plus/Plus

Query  1    GGTTTATTTGCAGCGTTGTTTG  22
            |||||||||||||| || ||||
Sbjct  154  GGTTTATTTGCAGCATTATTTG  175
>IPCPKDNE_01849 Bicarbonate transporter BicA [GCF_000350105.1
d__Bacteria;p__Cyanobacteria;c__Cyanobacteriia;o__Cyanobacteriales;f__Nostocaceae;g__Richelia;s__Richelia
intracellularis]
Length=273

 Score = 41.9 bits (45),  Expect = 0.012
 Identities = 24/25 (96%), Gaps = 0/25 (0%)
 Strand=Plus/Minus

Query  1    CACCAGTCATGATAACAGTCATTGG  25
            ||||||||||||| |||||||||||
Sbjct  238  CACCAGTCATGATGACAGTCATTGG  214
```

Fig. S10 (continued). **(C)** BlastN alignments for oligonucleotides designed to detect RintRC01_3892 as queries against RintRC_4851 (top) and RINTHH_3990 (middle, *bicA*RC82-F; bottom, *bicA*82-R). Sequence similarities are shown in Fig S7B.

Fig S11. Overview of the inorganic C acquisition in the two diatom-*Richelia* symbioses. *Richelia intracellularis* resides in the periplasm of the *Rhizosolenia cleveii* diatoms (top), and *Richelia euintracellularis* lives as a true endobiont in the cytoplasm of *Hemiaulus hauckii* diatoms (bottom) (7,8). The concentrations of inorganic carbon (CO_2, HCO_3^-) vary within diatoms, and results from the passive diffusion (black arrows) and active transport (blue arrows) via the hypothesized 'chloroplast pump' mechanism. Predicted concentrations of CO_2 and HCO_3^- shown as previously described (2), and concentrations only shown in *R. cleveii* for simplicity; the same concentrations are presumed here for *H. hauckii.* Carbonic anhydrases (CA) also function in conversion of inorganic carbon; the CCM in DDAs is unknown, and presumed to function similar to that shown which is based on model diatoms (2). As presented in this work, a SulP family BicA transporter was identified for RintRC01, with an expected $K_{0.5}$ for bicarbonate of about 464 μM (periplasm concentration similar to that in the extracellular medium, about 2 mM), whereas no evidence for a BicA transporter for the true endobiont ReuHH01 was obtained. Combined our results suggest that the cellular location of the *Richelia* endobionts influences both the function and affinity for inorganic carbon transport. Also shown for *H. hauckii-R. euintracellularis* is the recent demonstration for the role of organic carbon (i.e., sugars) supplied by the host diatoms via an ABC transporter (GlsR, substrate binding protein) and a neutral invertase (InvB) (42). Previous works have shown presence of membrane vesicles formed on the cell envelope of both *Richelia* which could potentially function in metabolite transfer between partners (7,8). The following abbreviations apply: pyr, pyrenoid, CER/PPC, chloroplast endoplasmic reticulum/periplastidial compartment.

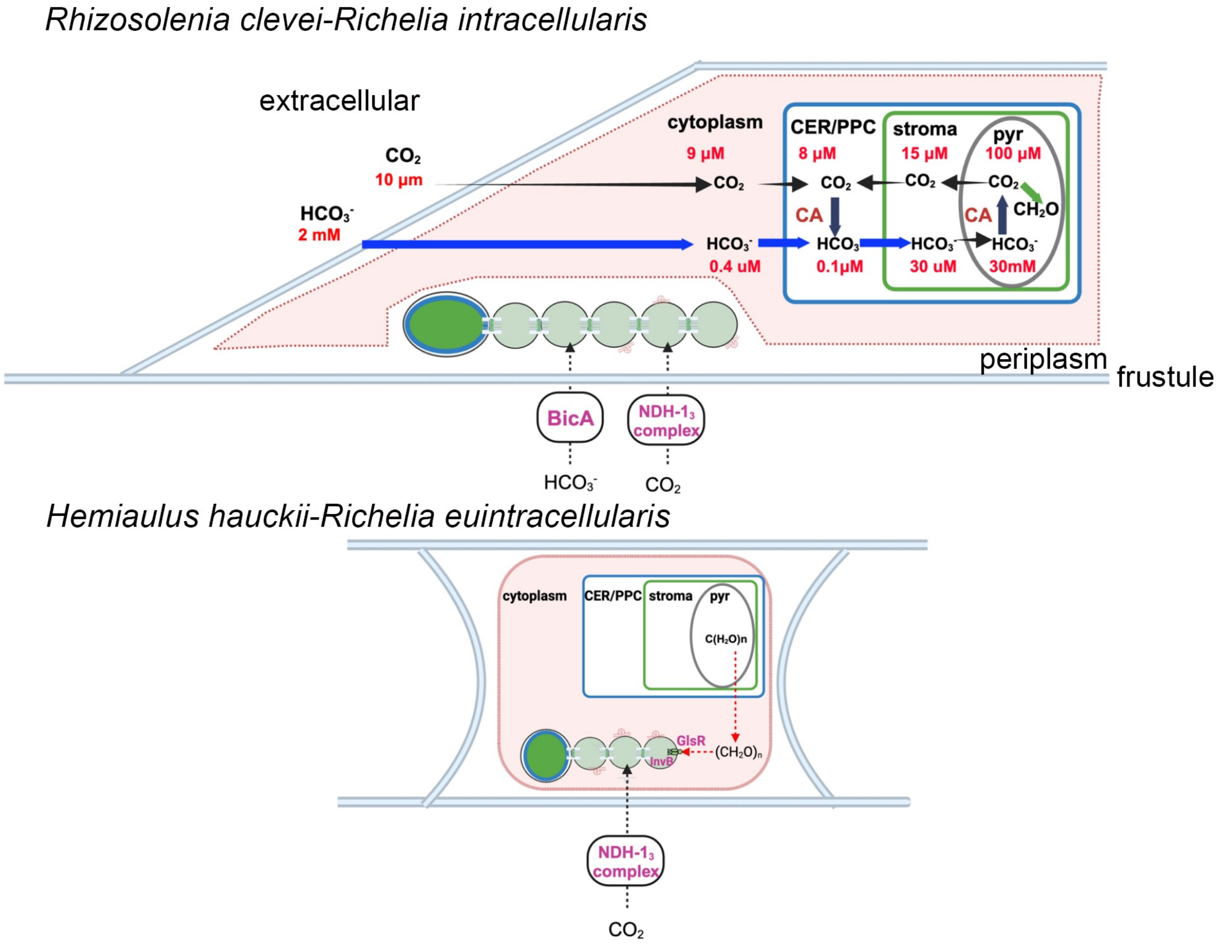

Table S1. List of the oligonucleotides used in the construction of plasmids containing the symbiotic *Richelia* genes. Introduced restriction enzyme cutting sites are underlined.

Primer name	Sequence (5´ to 3´)
RintRC_3892-5 (ClaI & NdeI sites)	GGCATCGATAAGGAATTATAACATATGCAACTTTTGAATCGCATCCAC
RintRC_3892-6 (PvuI & SphI sites)	AAACGATCGAAGCATGCTCTGACTATGAGTTTGTGGGCA
RintRC_3892-2	CAGGTGAATCATCTATGGGTGC
RintRC_3892-7	CCGATACCCGACATGAAGC
RintRC_4851-1	ACGTGAGGCGGAAACTCCAA
RintRC_3409-1	AAGGCGGTCAAAACAGCGAT
RintHH_3990-1	AATGCCATTGCCACGCTACT
RintHH_20770-1	CAAAATGTCGGCTCTCGCGT
PcpcB560_F	ATCGAATTCCACCTGTAGAGAAGAGTC
5_nrsD-F (BssHI site)	AAGCGCGCCTTTCACTGCTTGCGGAACC
RC-2	CAGGTGAATCATCTATGGGTGC

Table S2. Summary of oligonucleotides used in the RT-qPCR assays. RintRC_3892, RintRC_4851, RintRC_3409, RintHH_20770, RintHH_3990-60 were designed to amplify the SulP-like proteins from *Richelia* spp, and RintRC_6009 was designed to amplify the *secA* gene (used as a control). Sequences were taken from RintRC_01 draft genome (Genbank Assembly: GCA_ 000613065.1). The *bicA* and *secA* genes from *Synechocystis* WT were taken from the *Synechocystis* genome (Genbank Assembly: GCF_000009725.1).

Target	Forward 5' to 3'	Probe 5' to 3'	Reverse 5' to 3'
RintRC_3892	GGTTTATTTGCAG CGTTGTTTG	TACACCCACCCTGATT TCCGAACCTACC	CACCAGTCATGATAACAGTCA TTGG
RintRC_4851	GCTTCATGTCTG GGATTG	TACCTTGGCTGAACTG GGATGTCCC	GGATTGGGAGTAGTGAGATA
RintRC_3409	TGTTGGCTTCCTT CCTTAC	TGGATGAGCTTACCGA CACACCCA	TGGCAATACCTTGACCTTTA
RintHH_20770	GTTTACCCTGCCT GAAGTA	TCTTTGACATTAGCAAC CGTCGGCT	AGAAGAGGTGAGGAAAGAAG
RintHH_3990-60	GTCAGGACATTG CTAACTTAAT	TAGTTGCATCTGCACC AGGTAGACCC	CTACGAGTTACTCCTGATAGT G
RintRC_6009	GAAGGTGGAGTG ATGGATTA	ATTCAGCCGGAAACCC AAACCCTA	TCGGATACAGCAAGAATAGG
WT_bicA_sll0834	GACCCGCACAGA ACATAA	ACATTACATTGCCGAT GCCCTGGC	CCCAAGCCACCAAATAAAC
WT_secA_sll0616	AAAGCGGTAGCA TTGGA	TGCGAAGAATTGCACC AACAGGGT	GCAACAGTCGGGAAATAAC

Table S3. Summary of results from the RT-qPCR assays to estimate the expression of RintRC_3892 and RintRC_4851. Expression was normalized to *secA* (RintRC_6009) expression. Details on the sample location, time of sampling, and normalized cDNA L^{-1} are provided; samples that were below detection are noted as bd, samples noted as detected but not quantifiable (dnq) indicate the samples which had 1 or 2 of the 3 replicates that amplified, samples not run are noted as nr; The ocean basin use the following abbreviations: NA or SA, North or South Atlantic; SCS, South China Sea.

Sample ID	Station	Longitude	Longitude	Ocean Basin	Local sampling time	Depth (m)	Normalized cDNA RintRC_3892 L^{-1}	Normalized cDNA RintRC_4851 L^{-1}
681	1	15º 30’ N	21º 29’ W	NA	10:14	5	bd	bd
682	1				10:14	40	bd	bd
683	1				10:14	50	bd	bd
695	3	9º 29.95’ N	22º 0.04’ W	NA	23:35	5	4.34	0.30
696	3				23:35	20	bd	bd
697	3				23:35	42	bd	bd
714	6	0º 0.45´ N	21º 59.23´W	NA	23:40	5	5.15	0.2
715	6				23:40	15	4.36	0.51
716	6				23:40	30	dnq (2)	bd
723	6				11:00	5	31.35	nr
724	6				10:05	20	13.15	nr
725	6				10:05	35	9.08	nr
731	7	1º 59.42´ S	21º 59.77´W	SA	23:10	5	2.54	0.91
732	7				23:10	20	2.20	2.81
733	7				23:10	40	bd	bd
608	17	10 º59.70’ N	55º 26.78’ W	NA	17:40	5	8.66	2.03
609	17	17			17:40	20	dnq (2)	dnq (1)
610	17	17			17:40	40	bd	bd
10129	3	12º 39.04’N	109º48.280’E	SCS	8:40	0	bd	bd
10062	6	11º 8.439’N	109º37.036’E	SCS	4:05	45	bd	bd
10063	6				4:05	30	bd	bd
10064	6				4:05	20	11.15	dnq(1)
10065	6				4:05	13	6.10	6.37
10066	6				4:05	1	dnq(1)	nr
10165	9	11º 8.439’N	109º 37.062’E	SCS	12:30	8	3.21	dnq(1)
10166	9				12:30	5	bd	nr
10171	10	9º 51.2082´N	107º 0.558’E	SCS	3:55	1	5.82	4.17
10203	17	9º44.5968’N	108º 40.448’E	SCS	3:40	25 (1.5L)	3.80	3.94
